# Pharmacological Tuning of Adenosine Signal Nuances Underlying Heart Failure With Preserved Ejection Fraction

**DOI:** 10.3389/fphar.2021.724320

**Published:** 2021-08-20

**Authors:** Alexandrina Campos-Martins, Bruno Bragança, Paulo Correia-de-Sá, Ana Patrícia Fontes-Sousa

**Affiliations:** ^1^ Laboratório de Farmacologia e Neurobiologia, Centro de Investigação Farmacológica e Inovação Medicamentosa (MedInUP), Instituto de Ciências Biomédicas Abel Salazar, Universidade do Porto (ICBAS-UP), Porto, Portugal; ^2^ Department of Cardiology, Centro Hospitalar Tâmega e Sousa, Penafiel, Portugal

**Keywords:** adenosine, adenosine receptor, preserved ejection fraction heart failure, cardiac comorbidities, cardiac fibrosis and hypertrophy, endothelial dysfunction

## Abstract

Heart failure with preserved ejection fraction (HFpEF) roughly represents half of the cardiac failure events in developed countries. The proposed ‘systemic microvascular paradigm’ has been used to explain HFpHF presentation heterogeneity. The lack of effective treatments with few evidence-based therapeutic recommendations makes HFpEF one of the greatest unmet clinical necessities worldwide. The endogenous levels of the purine nucleoside, adenosine, increase significantly following cardiovascular events. Adenosine exerts cardioprotective, neuromodulatory, and immunosuppressive effects by activating plasma membrane-bound P1 receptors that are widely expressed in the cardiovascular system. Its proven benefits have been demonstrated in preclinical animal tests. Here, we provide a comprehensive and up-to-date critical review about the main therapeutic advantages of tuning adenosine signalling pathways in HFpEF, without discounting their side effects and how these can be seized.

## Introduction

Heart failure (HF) is a clinical syndrome characterized by alterations in the cardiac structure and/or function; it is associated with a poor quality of life, high rates of hospitalizations, and significant mortality (Ponikowski et al., 2016). HF with preserved ejection fraction (HFpEF) is a subclass of HF defined by left ventricular ejection fraction equal to or above 50% (Ponikowski et al., 2016). Nowadays, it represents up to half of all cases of HF in the developed world, which at least in part may be explained by the current lifestyle and/or prolongation of the life expectancy (Dunlay et al., 2017). HFpEF has a “heterogeneous” presentation often coursing with unspecific clinical symptoms (e.g., exercise intolerance and dyspnoea), challenging its diagnosis and clinical management (Ponikowski et al., 2016). Multiple cardiac-related systemic comorbidities drive the molecular and hemodynamic mechanisms implicated in this disease. Emerging data support systemic microvascular inflammation as an enduring aggravation factor (Paulus and Tschope, 2013). These findings contrast with HF with reduced ejection fraction (left ventricular ejection fraction under 40%), where cardiomyocytes loss and their replacement by fibrotic tissue is the most frequent pathological hallmark (Simmonds et al., 2020). In this case, the systolic dysfunction results in neurohormonal activation of the renin-angiotensin-aldosterone system along with sympathetic stimulation, supporting the indication for using angiotensin-converting enzyme inhibitors, mineralocorticoid receptors antagonists and *ß*-blockers as disease-modifying drugs (Ponikowski et al., 2016). Conversely, there is still no proven therapy to impact the course of HFpEF, thus representing one of the biggest challenges of cardiovascular medicine nowadays (Iliesiu and Hodorogea, 2018). Therefore, unraveling the dysfunctional signalling pathways associated with HFpEF may shed some light to propose novel pharmacological therapies to this unmet clinical condition.

The purine nucleoside adenosine was first identified in 1929 when Drury and Szent-Gyorgyi successfully extracted a rhythm-influencing adenylic substance from the mammalian heart and other tissues (Drury and Szent-Gyorgyi, 1929). Only in 1972, Geoff Burnstock (born: May 10, 1929, died: June 2, 2020) coined the term purinergic signalling referring to the extracellular effects of adenosine 5′-triphosphate (ATP). Following Burnstock’s pioneering work on the role of ATP-sensitive P2 purinoceptors, its metabolite adenosine soon became recognized as an extracellular signalling molecule through the activation of plasma membrane-bound P1 receptors family that are expressed in every organ systems in the body. In the cardiovascular system, adenosine is considered a “retaliatory metabolite” because it originates from the catabolism of ATP released from stressed cells, thus contributing to decrease cellular energy consumption while favoring the blood tissue supply (Newby et al., 1990). Given this, adenosine-mediated signals have been implicated in many pathophysiological processes. Nowadays, adenosine receptor ligands are being extensively investigated as promising druggable compounds, some of which are undergoing clinical trials (Borah et al., 2019). However, besides the native nucleoside, only a limited number of adenosine-related drugs are approved for clinical use (Borah et al., 2019).

**FIGURE 1:**
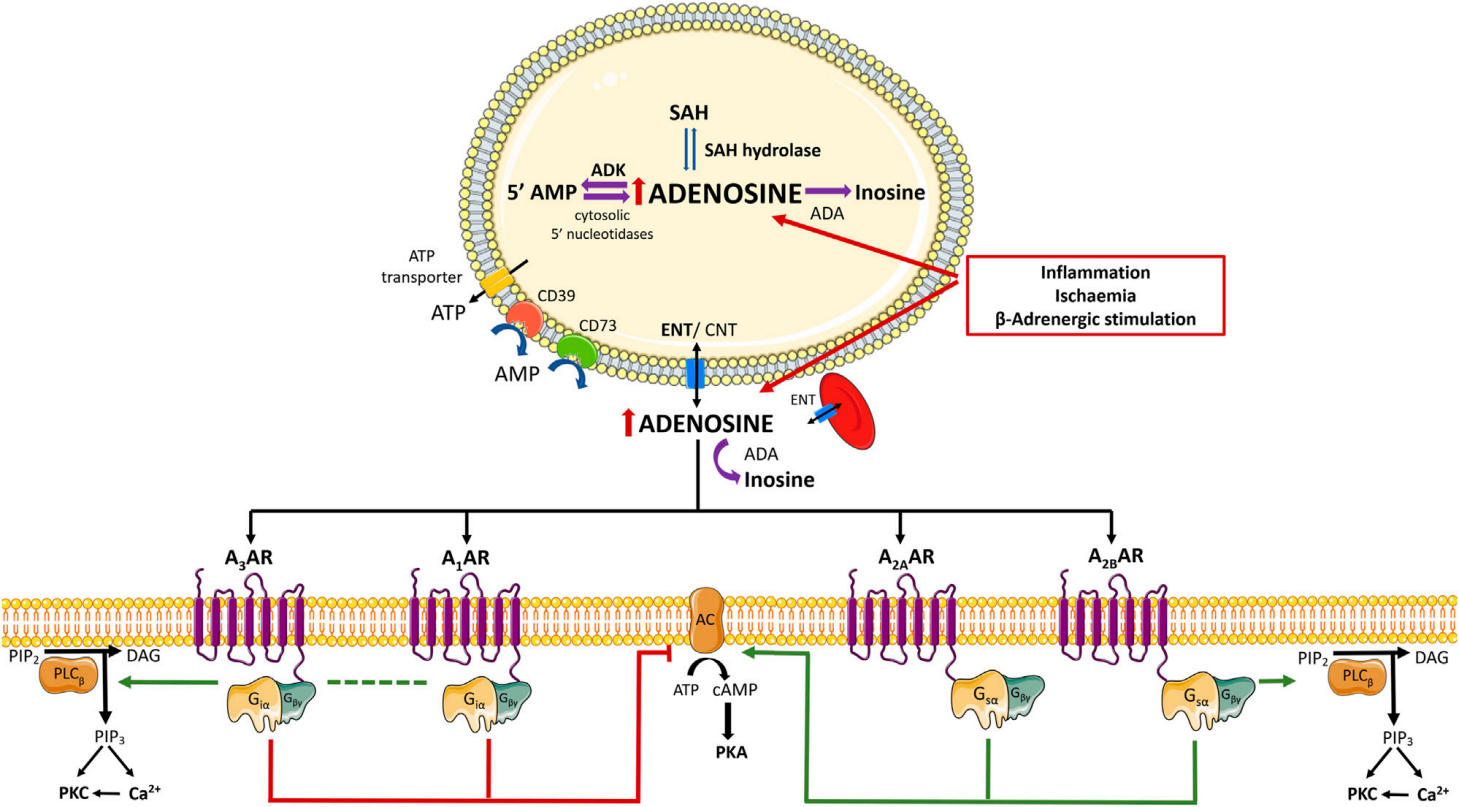
**Schematic representation of adenosine biosynthesis and complex signalling pathways induced by adenosine receptors activation.** Adenosine is a purine nucleoside continuously generated from the catabolism of adenine nucleotides via a cascade of nucleotidases. Regarding the fate of intracellular adenosine, it may (preferentially) re-enter the purines savage pathway by intracellular phosphorylation via adenosine kinase (ADK) or it is inactivated to inosine by adenosine deaminase (ADA). The main source of adenosine results from the hydrolysis of S-adenosylhomocysteine (SAH) by SAH hydrolase. Under low oxygen availability and/or during high energy working loads, recruitment from ATP hydrolysis increases favouring intracellular adenosine accumulation, which promotes its diffusion to the extracellular medium via equilibrative nucleoside transporters (ENTs). Extracellular adenosine may also result from the extracellular breakdown of released adenine nucleotides, namely adenosine 5′-triphosphate (ATP), ADP (adenosine 5′-diphosphate), and AMP (adenosine 5′-monophosphate), by a cascade of ecto-nucleotidases bound to the plasma membrane. CD39 dephosphorylates ATP directly into AMP; the rate limiting enzyme of the ecto-nucleotidase cascade is ecto-5′-nucleotidase/CD73, which dephosphorylates AMP to adenosine and inorganic phosphate. Extracellular adenosine levels are tightly regulated by cellular uptake via ENTs and/or by deamination into inosine by ADA. Adenosine activates 4 G protein-coupled receptor (GPCRs) known as P1 receptors (adenosine receptors (ARs): A_1_AR, A_2A_AR, A_2B_AR, A_3_AR). In brief, A_1_AR and A_3_AR are negatively coupled to adenylate cyclase (AC) through binding to G_i_ and G_o_ proteins, resulting in decreased intracellular cyclic AMP (cAMP) levels. Both A_2A_AR and A_2B_AR are coupled to G_s_ proteins and stimulate AC leading to increases in cAMP accumulation. Despite the canonical positive and negative coupling to the AC/cAMP system, A_1_AR, A_2B_AR, and A_3_AR are also entitled to activate phospholipase C-beta (PLC- β) isoform, resulting in increased inositol 1,4,5-trisphosphate (IP3) and intracellular Ca^2+^ mobilization; intracellular Ca^2+^ and diacylglycerol (DAG) production contribute to stimulate Ca^2+^-dependent protein kinase C (PKC) and/or downstream Ca^2+^-dependent pathways. Adapted from (Borea et al., 2018). Green arrows and red bars indicate the effects induced or blocked by adenosine receptor activation, respectively. Illustration used elements from Servier Medical Art (http://smart.servier.com). AC, Adenosine cyclase; ADA, Adenosine deaminase; ADP, 5′adenosine diphosphate; ADK, Adenosine kinase; AMP, 5′-adenosine monophosphate; AR, Adenosine receptor; ATP, 5′-adenosine triphosphate; cAMP, cyclic AMP; CNT, Concentrative nucleoside transporter; DAG, diacylglycerol; ENT, Equilibrative nucleoside transporter; GCPR, G protein-coupled receptor; IP3, inositol 1,4,5-trisphosphate; PKA, protein kinase A; PKC, protein kinase C; PLC- *ß*, Phospholipase C- beta; SAH, S-adenosylhomocysteine.

**FIGURE 2:**
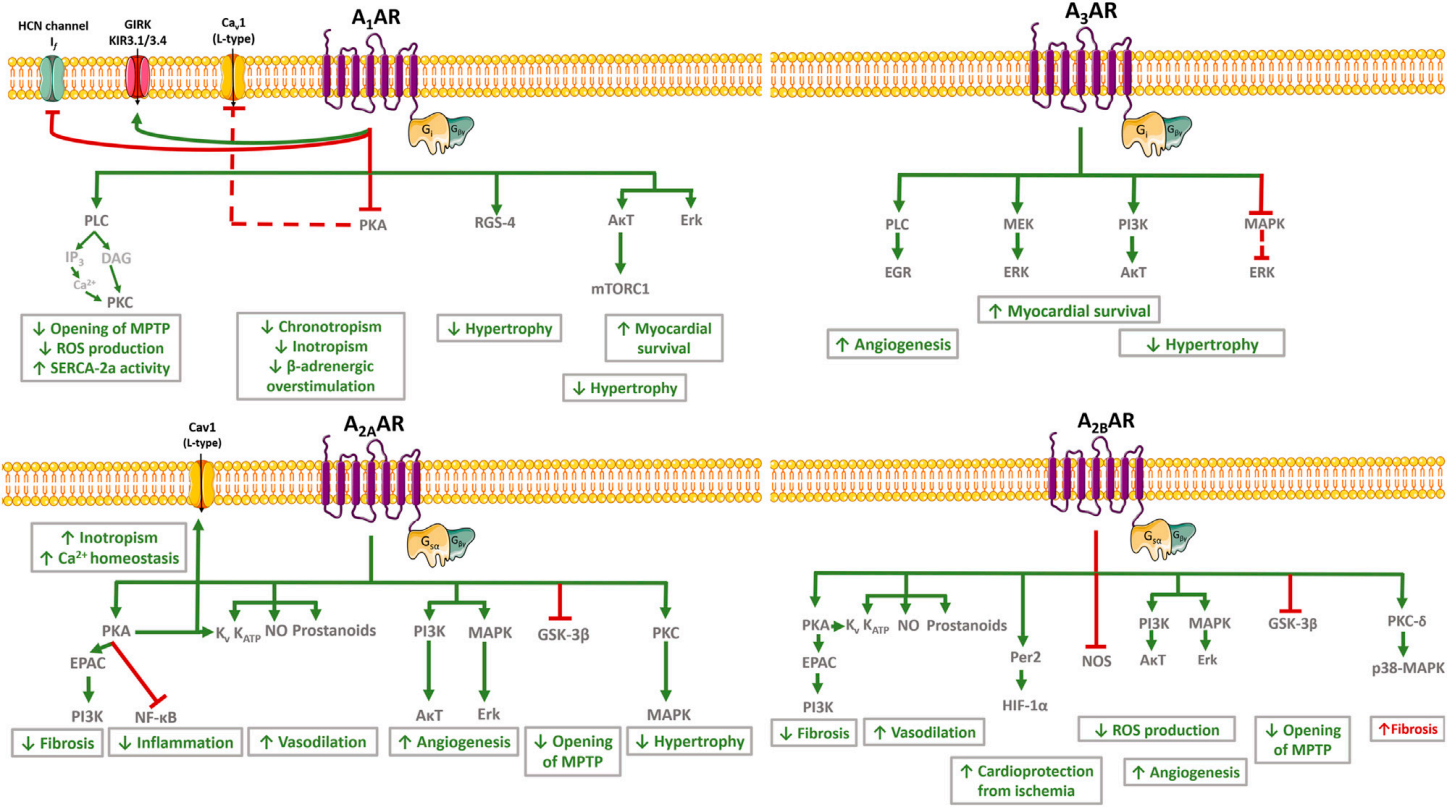
**Schematic representation of cardiovascular effects of adenosine and related signalling pathways.** In the cardiovascular system, adenosine exerts protective effects to control neuronal output (anti-adrenergic effect) and inflammation, while decreasing cardiac metabolism, myocardial contractility, impulse generation and conduction, and the coronary tone. These effects depend on the cellular type involved. The A_1_AR activation mediates cardioprotective effects by reversing cardiac hypertrophy/remodelling, improving mitochondrial function, enhancing SERCA2a activity and improving Ca^2+^ handling. The A_1_AR activation is also associated with an anti-ischemic effect by decreasing catecholamine release and by counteracting *ß*-adrenergic dysfunction. Activation of A_2A_AR attenuates cardiac inflammation, fibrosis and hypertrophy, while favoring vasodilation and angiogenesis. Despite the A_2B_AR-related vasodilation, angiogenesis and protection from ischemic preconditioning, conflicting evidence exist regarding the role of this receptor in fibrosis and related cardiac ischemic remodelling. Activation of A_3_AR is also associated with controversial results; this receptor mediates cardioprotective effects by increasing angiogenesis and myocardial survival during reperfusion, but it may be deleterious by aggravating cardiomyocytes hypertrophy. Green arrows and red bars indicate effects induced or blocked by adenosine receptors activation, respectively. The red dashed line indicates that the effect is indirectly blocked by adenosine receptors activation. Illustration used elements from Servier Medical Art (http://smart.servier.com) AR, Adenosine receptor; AκT, Protein kinase B; Ca_v_1 channel, L-type calcium channel subunit 1; DAG, diacylglycerol; EGR, Early Growth Response; EPAC, Exchange protein directly activated by cAMP; ERK, Extracellular signal-regulated protein kinase; GIRK and KIR3.1/3.4, G protein coupled inwardly rectifying K^+^ channels; GSK-3, Glycogen synthase kinase-3; HCN channels, Hyperpolarization-activated cyclic nucleotide-gated channel; HIF-1α, Hypoxia-inducible factor-alpha; IP_3_, Inositol 1,4,5-trisphosphate; I_*f*_ , “funny” hyperpolarization-activated current; K_ATP_, ATP-sensitive K^+^ channel; K_V_, voltage-gated K^+^ channels; MAPK, Mitogen activated protein kinase; MEK, Mitogen-activated protein kinase; MPTP, Mitochondrial permeability transition pores; mTORC1, Rapamycin complex 1; NF-κB, Nuclear factor-κB; NO, Nitric oxide; Per2, Period 2; PI3K, Phosphoinositol-3 kinase; PKA, Protein kinase A; PKC, Protein kinase C; PLC, Phospholipase C; ROS, Reactive oxygen species; SERCA2a, Sarco/endoplasmic reticulum Ca^2+^-ATPase 2a.

Current knowledge points towards adenosine as a ubiquitous signalling mediator of countless physiological processes throughout the body via the activation of 4 G protein-coupled receptors known as P1 receptors [adenosine receptors (ARs): A_1_AR, A_2A_AR, A_2B_AR, A_3_AR] (Borea et al., 2018). The A_1_AR and A_3_AR were first characterized as negatively coupled to adenylate cyclase (AC) through G_i_ and G_o_ proteins binding, which normally decrease intracellular cyclic adenosine 5′-monophosphate (cAMP) levels. Contrariwise, both high-affinity A_2A_AR and low-affinity A_2B_AR couple to G_s_ proteins and stimulate AC leading to intracellular cAMP accumulation. Despite this simplistic view of the canonical coupling of P1 receptors to the AC/cAMP pathway, evidence has been gathered demonstrating that A_1_AR, A_2B_AR, and A_3_AR may also activate the phospholipase C beta (PLC-β) isoform. Stimulation of PLC releases inositol 1,4,5-trisphosphate from the plasma membrane and trigger intracellular calcium (Ca^2+^) mobilization; both intracellular Ca^2+^ recruitment and diacylglycerol (DAG) production synergize to stimulate Ca^2+^-dependent protein kinase C (PKC) and/or other Ca^2+^-associated downstream pathways (Figure 1) (Borea et al., 2018). Interestingly, the cardiovascular (and many other) effects of adenosine are mediated by a multiplicity of more recently discovered intracellular signalling cascades, such as exchange protein directly activated by cAMP (EPAC), phosphatidylinositol 3-kinase (PI3K)/protein kinase B (Akt), mitogen-activated protein kinase (MAPK)/extracellular signal-regulated kinases (Erk), glycogen synthase kinase 3β (see *From Cardiovascular Effects of Adenosine to Cardioprotection* and Figure 2). Due to their relevance to the current knowledge, involvement of these pathways to adenosine-mediated effects will be further discussed in the following sections.

## Generation, Transport, and Metabolism of Adenosine

Adenosine is a purine nucleoside continuously generated from the catabolism of adenine nucleotides via a cascade of nucleotidases, including the rate-limiting enzyme 5′-nucleotidase, which dephosphorylates AMP to adenosine both intra- and extracellularly (Figure 1) (Rubio et al., 1973). Regarding the fate of intracellular adenosine, it may (preferentially) re-enter the purines salvage pathway through intracellular phosphorylation via adenosine kinase (ADK) (Km = 2 µM). Alternatively, the nucleoside can be inactivated to inosine by adenosine deaminase (ADA) (Km = 17–45 µM) when the phosphorylation pathway is overloaded or inhibited (Borea et al., 2018). Another possible source of adenosine comes from the hydrolysis of S-adenosylhomocysteine by S-adenosylhomocysteine hydrolase, being this the main pathway responsible for the intracellular levels of adenosine in conditions of adequate oxygen supply (Schrader et al., 1981). Under low oxygen supply conditions and/or during high energy working loads, ATP hydrolysis increases resulting in intracellular adenosine accumulation. As a consequence, adenosine diffuses to the extracellular milieu via equilibrative nucleoside transporters (ENTs), mostly ENT1 and ENT2 encoded by SLC29A1 and SLC29A2 genes, respectively (Governo et al., 2005; Dip, 2009). NBTI-sensitive (ENT1) and insensitive (ENT2) transporters are expressed in the vascular endothelium, erythrocytes, inflammatory cells, and cardiomyocytes (Eltzschig et al., 2005; Morote-Garcia et al., 2009). The sensitivity of both transporters to dipyridamole allowed this drug to be introduced in the market as a coronary vasodilator more than half a century ago, which is still used nowadays as antithrombotic and vasodilator with promising anti-oxidant properties (Ciacciarelli et al., 2015). Transport of adenosine across the plasma membrane can also occur through concentrative nucleoside transporters encoded by SLC28A1, SLC28A2, and SLC28A3 genes; these proteins concentrate adenosine (and other purines and pyrimidines) inside the cells via a Na^+^-nucleoside cotransporter (Pastor-Anglada and Perez-Torras, 2018). While ENTs may have a major role in maintaining nucleoside homeostasis, concentrative nucleoside transporters may contribute to adenosine sensing and signal transduction inside the cells (transceptor function).

As aforementioned, extracellular adenosine may result from the release of the nucleoside via ENTs and/or through the extracellular breakdown of released adenine nucleotides, namely ATP, ADP, and AMP, by a cascade of ecto-nucleotidases bound to the plasma membrane. Besides originating from damaged cells, adenine nucleotides may be released from intact cells by vesicular exocytosis, as well as via other mechanisms involving plasma membrane “pores”, namely ionotropic P2 purinoceptors, ABC proteins and hemichannels containing connexins and/or pannexins (Lazarowski et al., 2011).

Four members of the ecto-nucleoside triphosphate diphosphohydrolase (E-NTPDase) family (NTPDase1, 2, 3, and 8) and two members of the ecto-nucleotide pyrophosphatases/phosphodiesterases (E-NPP) family (NPP1 and NPP3) are located at the plasma membrane and hydrolyze extracellular nucleotides (Yegutkin, 2008). NTPDase1 (CD39 or apyrase) dephosphorylates ATP directly into AMP, with minimal accumulation of ADP. NTPDase2 (ATPase) is a preferential nucleoside triphosphatase that hydrolysis ADP 10 to 15 times less efficiently than ATP, leading to minimal AMP accumulation. NTPDase3 and NTPDase8 are functional intermediates between NTPDase1 and NTPDase2. NTPDases are considered potential therapeutic targets due to their role in coagulation, immune responses, vascular inflammation, and cancer (Vieira et al., 2014). Interestingly, NPP1 and NPP3 phosphorylated product (e.g., AMP) has a higher binding affinity to this enzyme than substrates do, leading to the inhibition of nucleoside 5′-monophosphate release (Vieira et al., 2014). The rate-limiting enzyme of the ecto-nucleotidase cascade is ecto-5′-nucleotidase/CD73, which dephosphorylates AMP to adenosine and inorganic phosphate. This enzyme is abundantly expressed in immune (e.g., T and B lymphocytes) and mesenchymal originated cells (Bono et al., 2015).

Extracellular adenosine levels are tightly regulated by cellular uptake via ENTs and/or by deamination into inosine by ADA; this enzyme can be found either on the cell surface (ecto-ADA) or as a soluble form after cleavage of its anchor to the plasma membrane (exo-ADA). Low levels of adenosine are normally found in biological fluids. Nevertheless, the extracellular concentration of the nucleoside increases in stressed cells following ischemia/reperfusion, inflammation, neuronal activation, and tissue damage (Sumi et al., 2010; Grenz et al., 2011; Idzko et al., 2014). Adenosine in the plasma has a short half-life due to its rapid inactivation by blood cells and by the vascular endothelium (Meyskens and Williams, 1971). Notwithstanding this, high plasma levels of adenosine have been detected in heart failure, both in humans (Funaya et al., 1997; Asakura et al., 2007) and animals (Correia-de-Sa et al., 2015). Moreover, the ecto-5′-nucleotidase/CD73 activity increases in the plasma and ventricular myocardium of chronic HF patients (Fujita et al., 2008). Together, plasma adenosine and ecto-5′-nucleotidase/CD73 activity may be reliable markers for the diagnosis of severity grade and follow-up of HF, even though adenosine levels are normally underestimated due to its extensive inactivation in the blood stream.

## Adenosine in the Cardiovascular System

### Distribution of Adenosine Receptors in the Cardiovascular System

The A_1_AR is the most expressed adenosine receptor subtype found in the intact myocardium and isolated cardiomyocytes, being particularly abundant in atria. The second most abundant receptor in the heart is the A_2A_AR, followed by A_2B_AR and A_3_AR, which exhibit low expression levels in the heart (Headrick et al., 2013). Nevertheless, one should remember that this pattern is mostly influenced by receptors expressed in cardiomyocytes, which occupy up to 85% of the mammalian heart volume (Zhou and Pu, 2016). Looking more deeply into the distribution of adenosine receptors in cardiac cells, this expression profile changes with A_2B_AR and A_2A_AR being more abundant in endothelial cells and cardiac fibroblasts (Headrick et al., 2013). Other tissues and organs influencing the cardiac function are also enriched in adenosine receptors. Large amounts of A_1_AR are found in kidneys, adipose tissue, pancreas, and brain. The A_2A_AR is highly expressed in peripheral immune cells (particularly in leucocytes), platelets, smooth muscle fibers and endothelial cells (Headrick et al., 2013).

The activity of the low-affinity A_2B_AR has been physiologically neglected mostly because higher amounts of adenosine are required to activate this receptor even in cells where its expression is high (Sun and Huang, 2016). However, this assumption is changing as A_2B_AR over-functioning has been gathered in several pathological conditions, including hypoxia, inflammation, and cell stress, thus providing support for a meaningful role of this receptor in health and disease (Bessa-Goncalves et al., 2018). Considering the retaliatory nature of adenosine, the A_2B_AR can be seen as a “dormant” receptor that “wakes up” following cells and tissue injury (Bessa-Goncalves et al., 2018). The presence of the A_3_AR in cardiomyocytes, vascular smooth muscle cells, and immune cells have been implicated in its cardioprotective role against ischemia, as well as in the control of blood vessels tone and remodelling (Headrick et al., 2013).

### From Cardiovascular Effects of Adenosine to Cardioprotection

In the cardiovascular system, adenosine exerts additional protective effects to control neuronal output and inflammation, while decreasing cardiac metabolism, myocardial contractility, impulse generation and conduction, and the coronary tone; the nucleoside is also involved in the control of adrenergic responsiveness and blood pressure (Figure 2) (Headrick et al., 2013).

Stimulation of A_1_AR induces negative chronotropism and dromotropism, as this receptor is highly expressed in the heart conduction system (sinoatrial and atrioventricular nodes, and the His-Purkinje network) (Headrick et al., 2013). In this regard, while adenosine exerts direct inhibitory effects on chronotropism and dromotropism in atrial cardiomyocytes, it also counteracts *ß*-adrenergic effects on impulse generation and contractility. The A_1_AR-induced hyperpolarization of the supraventricular tissue is accomplished by favoring outward potassium (K^+^) currents through opening G protein-coupled inwardly rectifying K^+^ channels (GIRK or KIR3.1/3.4), while counteracting adrenergic effects through inhibition of Ca^2+^ influx and/or attenuation of hyperpolarization-activated cyclic nucleotide-gated channel four mediating the pacemaker “funny” (I*f*) currents (Belardinelli and Isenberg, 1983; Belardinelli et al., 1995). These effects lead to bradycardia and atrial hypocontractility. In this respect, our group demonstrated that adenosine-induced negative atrial inotropism may be partially counteracted by multiple downstream intracellular pathways ending up to increase the time available for Ca^2+^ influx through Cav1 (L-type) channels (Braganca et al., 2016). Taken together, the electrophysiological properties of the A_1_AR justify the use of adenosine in the treatment of supraventricular tachycardia and to control the rate of ventricular contractions during atrial fibrillation (Savelieva and Camm, 2008; Lim et al., 2009).

Adenosine counteracts the sympathetic drive operated by catecholamines on cardiac *ß*-adrenoceptors by decreasing impulse generation and contractility via a mechanism involving both pre- and post-junctional effects. The positive inotropic action of *ß*-adrenoceptors may be directly controlled by adenosine via inhibition of cAMP production and protein kinase A (PKA) activation (Dobson, 1983; Romano and Dobson, 1990). The A_1_AR-mediated cardioprotection extends to intracellular organelles, namely mitochondria through a PKC-mediated reduction of the permeability of mitochondrial transition pores, stabilization of mitochondrial membrane potential and inhibition of hypoxia-induced production of reactive oxygen species (ROS), which ends up in the opening of K_ATP_ mitochondrial channels and cytoprotection (Xiang et al., 2010a). Activation of A_1_AR also increases epidermal growth factor receptor activation through the action of myocardial survival kinases Erk 1/2, Aκt (Williams-Pritchard et al., 2011). Overall, these properties confer myocardial protection under ischemic pre-conditioning and ischemia/reperfusion situations (Reichelt et al., 2005; Morrison et al., 2006; Greene et al., 2016).

While myocardial actions of adenosine typically focus on activation of the most abundant A_1_AR, increasing data also suggest the involvement of the A_2A_AR in myocardial contractile performance (Dobson and Fenton, 1997) via PKA-dependent increases in intracellular Ca^2+^ (Dobson et al., 2008). The A_2A_AR counteracts the antiadrenergic effects of the A_1_AR at the intracellular signaling level, a phenomenon that is further amplified by the formation of A_1_AR:A_2A_AR heteromers (Fenton and Dobson, 2007). Both A_2A_AR (Iwamoto et al., 1994; Shryock et al., 1998) and A_2B_AR (Kusano et al., 2010) have vasodilatory effects mediated by cAMP and PKA activation in vascular smooth muscle cells (Iwamoto et al., 1994). Involvement of K_v_ and K_ATP_ channels (Berwick et al., 2010), nitric oxide (NO) (Li et al., 1995; Li et al., 1998), and prostanoids (Faria et al., 2006) production have also been demonstrated. Coupling of A_2A_AR and A_2B_AR to such downstream signaling pathways entitles adenosine to regulate coronary blood flow and to protect against chronic myocardial ischemia following endothelial dysfunction. Long-term stimulation of these receptors also fosters mitogenic signaling pathways resulting in vascular overgrowth and angiogenesis; these pathways include A_2A_AR and A_2B_AR mediated-PI3K/Aκt activation and MAPK/Erk activation by A_2A_AR (Ahmad et al., 2013; Du et al., 2015).

Mounting evidence has been gathered suggesting that A_2A_AR activation is cardioprotective and immunosuppressant during early reperfusion after myocardial infarction (MI). The selective A_2A_AR agonist, CGS 21680, decreased neutrophils adhesion to the endothelium and subsequent myocardial infiltration, together with a reduction of superoxide production, in a canine model of ischemia and reperfusion (Jordan et al., 1997). Moreover, another A_2A_AR agonist, ATL146e, attenuated resident mast cells degranulation in a mouse model of myocardial ischemia (Rork et al., 2008). Cardioprotection is also accomplished because activation of A_2A_AR prevents the infarct-boosting effect of interferon-gamma (IFN-γ) produced by CD4^+^ T cells, as this cytokine favors reperfusion injury by activating pro-inflammatory macrophages (Yang et al., 2006). Although preconditioning is hardly feasible in clinical settings, clinical trials are ongoing to test whether A_2A_AR agonists can be used in coronary artery disease and MI (Borah et al., 2019). In this context, it is worth noting that both adenosine and the selective A_2A_AR agonist, regadenoson, have been clinically approved for myocardial perfusion imaging (Cury et al., 2014).

To our knowledge, A_2B_AR is the only adenosine receptor subtype upregulated in ischemic hearts of both mice and humans (Gile and Eckle, 2016). Activation of the A_2B_AR controls Period 2 protein, a metabolic master-switch of myocardial adaptation to ischemia. This protein stabilizes the hypoxia-inducible factor 1α and, thereby, the transduction of glycolytic enzymes (Eckle et al., 2012) to optimize oxygen consumption and to protect the myocardium from infarction reperfusion (Gile and Eckle, 2016). Moreover, both A_2B_AR and A_2A_AR are associated to inhibition of mitochondrial glycogen synthase kinase 3β (GSK-3β) phosphorylation, thus preventing the mitochondrial permeability transition pore opening, which is a critical step to afford cardioprotection during myocardial reperfusion (Xi et al., 2009). Inhibition of ROS production by mitochondria broadens the cardioprotective effects of the A_2B_AR in cardiac ischemia by involving multiple PKC-mediated signalling pathways affecting nitric oxide synthase (NOS), PI3K/Aκt, and Erk 1/2 enzymatic activities (Kuno et al., 2007; Yang et al., 2011; Seo et al., 2015).

Only a few studies have been conducted to elucidate the role of the A_3_AR in cardiac pathophysiology, most probably because this receptor subtype has low expression levels in cardiac tissues. This situation may also occur because the A_3_AR has unpredictable effects in rodent models, as it behaves like a low affinity receptor for adenosine contrary to the high affinity for the nucleoside shown in human tissues (Headrick et al., 2013). Current data suggest that the A_3_AR limits injury processes occurring in the ischemic myocardium, while exerting an anti-inflammatory action during cardiac reperfusion (Ge et al., 2010). Surprisingly, A_3_AR agonists cause a biphasic hemodynamic response that is characterized, initially, by indirect activation of high affinity A_2A_AR followed by subsequent A_3_AR-mediated effects that prevail after A_2A_AR become desensitized (Tian et al., 2015a). Both in cardiomyocytes and in intact hearts of rats subjected to ischemia and reperfusion, A_3_AR agonists reduced the infarct size. This effect is related to the upregulation of pro-survival signaling pathways, such as mitogen-activated protein kinase 1/2- Erk 1/2 and PI3K/Aκt, which are known to decrease the activity of caspase-3 used as a biomarker of cellular apoptosis (Hussain et al., 2014). The cardioprotection associated with the A_3_AR activation may also be due to the induction of human coronary smooth muscle cells proliferation via a mechanism involving phospholipase C and downstream transcriptional factors activation, like the early growth response element 2/3 (Hinze et al., 2012).

## Impact of Adenosine in the Molecular Pathways Underlying HFpEF

### Systemic and Cardiac Inflammatory Breakthrough to Cardiac Fibrosis

**FIGURE 3:**
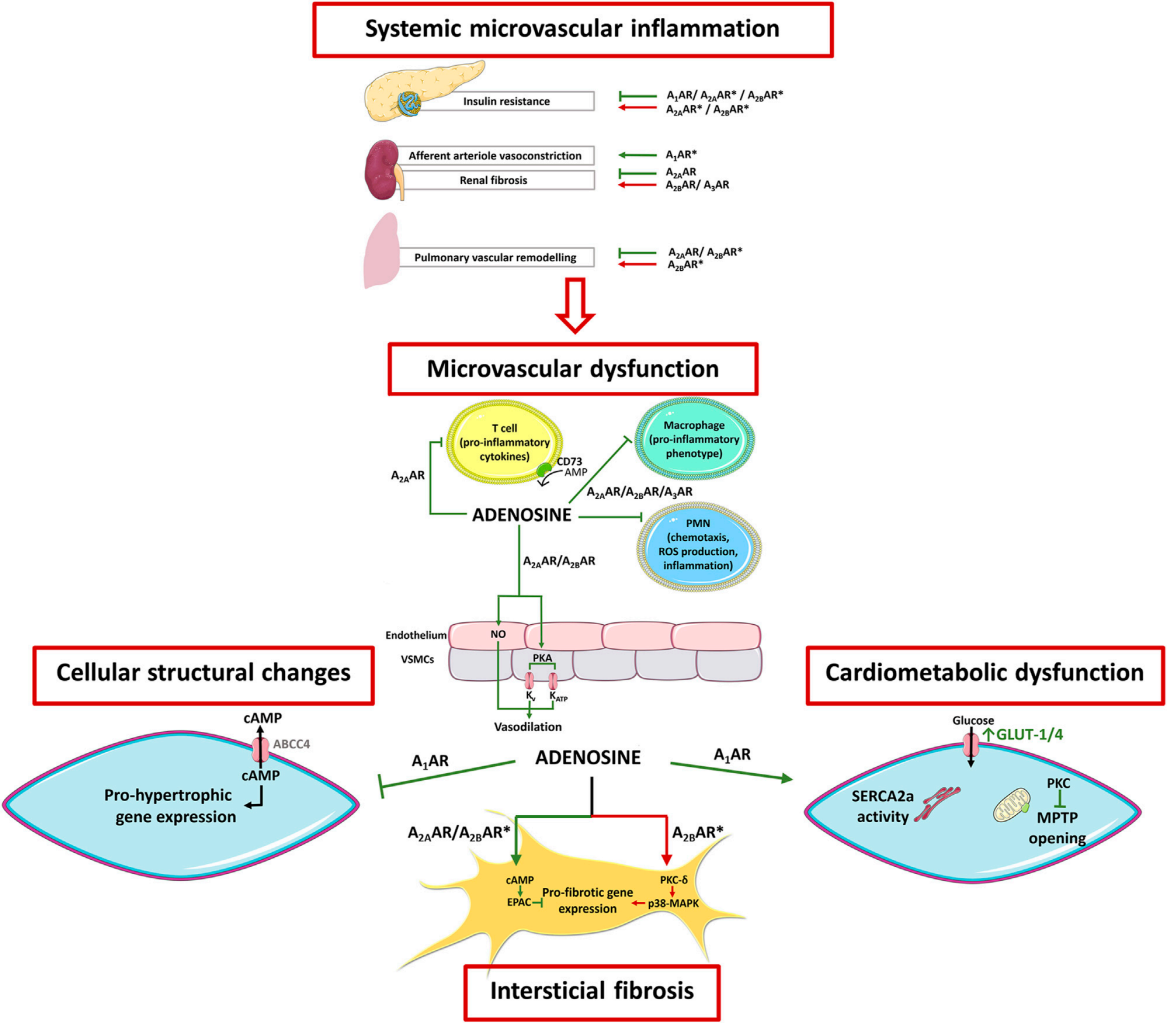
**Schematic representation of adenosine receptors involvement in HFpEF pathophysiology.** HFpEF is a complex clinical syndrome where comorbidities-induced systemic inflammation predispose and perpetuate microvascular dysfunction, as well cardiac structural and metabolic abnormalities. Overall, adenosine counteracts most of the pathophysiological features of this syndrome. These include 1) cardiac inflammation and microvascular dysfunction (via A_2_AR activation), 2) myocardial structural abnormalities (via A_2_AR and A_1_AR activation), and 3) energy metabolism and calcium handling (via A_1_AR activation). Conflicting evidence, however, exist regarding A_2B_AR-mediated cardioprotection, as this receptor has been implicated in both pro- and anti-fibrotic effects in the heart and lungs. Adenosine receptors also play important roles in cardiometabolic comorbidities related to HFpEF. Activation of A_1_AR improves the metabolic profile and induces renal afferent arteriole vasoconstriction, which can be protective when preservation of the glomerular architecture and function is needed. Stimulation of A_2A_AR and the A_2B_AR reduce lipolysis, but this beneficial effect may be partially counteracted by their action on skeletal muscles that contribute to insulin resistance. Activation of A_2A_AR counteracts renal damage due to its ability to reduce fibrosis, independently of A_3_AR and A_2B_AR are active or not. The arrows and bars indicate the effects induced or blocked by adenosine receptor activation, respectively. Green = beneficial effect; Red = deleterious effect; Asterisk (*) = contradictory/conflicting data. Adapted from Lam et al., 2018 and Headrick et al., 2013. Illustration used elements from Servier Medical Art (http://smart.servier.com) ABCC4, ATP-binding cassette sub-family C member 4; AR, Adenosine receptor; cAMP, cyclic adenosine monophosphate; EPAC, Exchange factor directly activated by cAMP; FFA, Free fatty acid; GLUT, Glucose transporter; MAPK, Mitogen activated protein kinase; MPTP, Mitochondrial permeability transition pores; NO, Nitric oxide; PKA, Protein kinase A; PKC, Protein kinase C; PMN, Polymorphonuclear leukocytes; SERCA2a, Sarco/endoplasmic reticulum Ca^2+^-ATPase 2a; *a*-SMA, Alpha-smooth muscle actin; VSMC, Vascular smooth muscle cell.

HFpEF is associated with systemic inflammation in its genesis that is evidenced by increased circulating levels of pro-inflammatory markers, such as interleukin-6 (IL-6) and tumor necrosis factor-α (TNF-α) (Kalogeropoulos et al., 2010). These findings support the “systemic microvascular paradigm” regarding HFpEF in which comorbidities-induced systemic inflammation predisposes and perpetuates dysfunction of the microvasculature (Figure 3) (Paulus and Tschope, 2013). The most important comorbidities associated with this systemic inflammatory state are diabetes mellitus (DM), overweight/obesity, hypertension, chronic obstructive pulmonary disease, and chronic kidney disease (Paulus and Tschope, 2013). Thus, the paradigm of HFpEF has been shifted from the traditional “overload model” to the revolutionary “microvascular hypothesis”, where patients are more easily identified by elevated body mass index rather than by elevated blood arterial pressure (Rozenbaum et al., 2019).

Supporting the concept of cardiac inflammation, left ventricular endomyocardial biopsies from patients with HFpEF show increases in the expression of biomarkers of inflammation and fibrosis, such as vascular cell adhesion molecule-1 (VCAM-1), CD3, CD11, and CD45-positive myocardial leucocytes, transforming growth factor-β (TGF-β), types I and III collagen species and extracellular matrix deposition (Westermann et al., 2011). Unbalanced cardiac inflammation causes several of the pathological features seen in HFpEF, such as endothelial dysfunction, fibrosis, concentric hypertrophy, and cardiometabolic functional abnormalities (Lam et al., 2018). Cardiac remodeling lead to left ventricular diastolic dysfunction (Westermann et al., 2011), which ultimately may cause unspecified clinical manifestations related to abnormal haemodynamics (e.g., dyspnoea and fatigue) that are characteristic of HFpEF (Lam et al., 2018).

The pleiotropic actions of adenosine in several organic systems put this retaliatory metabolite in good position to participate in most (if not all) the pathophysiological mechanisms associated with HFpEF. These include the control of systemic microvascular inflammation and related multi-organ comorbidities, in parallel to the nucleoside effects on cardiovascular function and myocardial remodelling. Adenosine regulates the activity of the immune system via self-contained signalling pathways aimed at promoting tissues integrity by resolving inflammatory insults (Figure 3), although a pro-inflammatory activity has also been described (reviewed in (Antonioli et al., 2014; Antonioli et al., 2019)).

Activation of the A_2A_AR is paramount to the anti-inflammatory effect of adenosine; the coupling of the A_2A_AR to the canonical AC/cAMP/PKA pathway downstream decreases nuclear factor-kappa B signalling and the release of pro-inflammatory cytokines (Sands et al., 2004). Adenosine signalling in neutrophils either stimulates the expression of adhesion molecules via A_1_AR or decreases adhesion of these cells to the vascular endothelium, as well as the production of superoxide radicals by A_2A_AR, A_2B_AR and A_3_AR activation. Anti-inflammatory (M2) macrophages overexpress A_2A_AR, A_2B_AR and A_3_AR, which activation attenuates the production of pro-inflammatory mediators (TNF-α, IL-6, IL-12, NO, and macrophage inflammatory protein-1α). Activation of A_2A_AR and A_2B_AR also favors 1) the release of the anti-inflammatory cytokine IL-10 by monocytes and macrophages (Antonioli et al., 2014; Antonioli et al., 2019), and 2) the switch from M1 (pro-inflammatory) to M2 (anti-inflammatory) macrophage phenotypes (Grinberg et al., 2009). Adenosine modulates lymphocyte functions on adaptive immunity, mainly through A_2A_AR activation and related suppressive effects, as it inhibits both IL-4 and IFN-γ production by naïve CD4^+^ T cells and Th1 and Th2 cells (Antonioli et al., 2014; Antonioli et al., 2019). Taken together, increased extracellular adenosine levels caused by inflammatory processes (Grenz et al., 2011; Idzko et al., 2014) emerges as a putative therapeutic target against inflammatory diseases with encouraging results in preclinical settings (Antonioli et al., 2014). The analogy to clinical data concerning the adenosine actions in rheumatoid arthritis (Silverman et al., 2008) and cancer immunotherapy (Antonioli et al., 2017), also supports this idea. In this context, adenosine mediates the anti-inflammatory effects of methotrexate, a gold standard treatment for patients with rheumatic diseases. By inhibiting certain enzymatic pathways, methotrexate induces adenosine release allowing the nucleoside to exert its anti-inflammatory effects via A_2A_AR and A_3_AR activation (Chan and Cronstein, 2010).

Mice submitted to transverse aortic constriction (TAC), a model of HFpEF, show significant cardiac inflammation that is initiated by transient myeloid cells infiltration followed by a chronic increase of myocardial T cells (Table 1) (Quast et al., 2017). Noteworthy, chemotaxis and activation of myocardial T cells are under the immunosuppressive control of adenosine formed by ecto-5′-nucleotidase/CD73 (Eltzschig et al., 2004) and A_2A_AR activation (Hamad et al., 2012). Indeed, the production of inflammatory cytokines (IL-3, IL-6, IL-13, IL-17, macrophage inflammatory proteins 1α and 1β) associated with cardiac fibrosis and decreased contractility is exaggerated by TAC in mice lacking CD73 on T cells, as well as in global CD73 mutants (Quast et al., 2017). These animals exhibit compensatory increases in the enzymatic machinery participating in adenosine formation (e.g. CD39, pyrophosphatases ENPP1 and ENPP3, and CD38) and overexpress A_2A_AR in activated T cells infiltrating the injured heart (Quast et al., 2017). Additionally, overexpression of A_2A_AR attenuates cardiac inflammation and remodelling, thereby attenuating cardiac dysfunction (Table 1) (Hamad et al., 2012). These data provide compelling evidence that deficient adenosine formation by T cells lacking CD73 worsens the HFpEF. This raised the hypothesis that increasing extracellular adenosine formation from the extracellular catabolism of released adenine nucleotides and/or direct activation of the A_2A_AR subtype may overcome HFpEF by reducing myocardial damage caused by pro-inflammatory cytokines and maladaptive cardiac remodelling (Hamad et al., 2012; Quast et al., 2017). Thus, maintenance of ecto-5′-nucleotidase/CD73 activity may be crucial to define the pattern of extracellular ATP-derived adenosine formation favoring A_2A_AR activation to prevent the pathological changes observed in HFpEF, as also predicted in other cell systems (Oliveira et al., 2015).

**TABLE 1:** Results of adenosine receptors agonism/antagonism in heart failure with preserved ejection fraction and pulmonary hypertension. Different agonists and antagonists were used to assess adenosine effects on cardiovascular diseases. The grey area corresponds to studies carried out in human patients.

	Adenosine receptors	Adenosine receptor modulation			Model and species	Outcome	Comments	Refs
		Pharmacological tool						
		Agonist	Antagonist	Other				
Heart failure with preserved ejection fraction				Dipyridamole (Adenosine uptake blocker)	Sprague–Dawley rats (abdominal aortic banding)	↑ Myocardial adenosine levels	A_1_AR^b^ desensitization is in line with an increased exposition to adenosine *in vivo*	Chung et al. (1998)
						↓ Chamber dilatation, LV^a^ filling abnormalities and pulmonary congestion		
						Prevented *ß*-adrenergic desensitization		
				CD73 KO^c^ and CD4 CD73 KO	Mice (transverse aortic constriction)	↑ CD73 exclusively on T cells	Transient ↑ in infiltration of monocytes, granulocytes, and B cells and persistent ↑ of cytotoxic T cells, T-helper cells, and regulatory T cells	Quast et al. (2017)
						↑ T cell enzymatic machinery for formation of AMP^d^		
						↓ Cardiac function and ↑cardiac fibrosis in both CD73 KO and CD4 CD73 KO mice		
						↑ Proinflammatory cytokines in CD73 KO mice		
						↑A_2A_AR on T cells		
				CD73-KO	Mice (transaortic constriction)	↑ Myocardial hypertrophy	Cardiac dysfunction was not present in healthy animals. Attenuation of myocardial hypertrophy is due to mTOR^e^/p70S6K activation	Xu et al. (2008)
						↑ LV dilation and LV dysfunction		
				ADK^f^-KO	Mice (transaortic constriction)	↑ LV hypertrophy and dysfunction	In rat neonatal cardiomyocytes, ADK activity dampened cardiac growth signalling and excessive microtubule stabilization/detyrosation	Fassett et al. (2019)
						↑ Pulmonary congestion		
	A_1_AR	CADO^g^ (non selective)			C57BL/6 mice (transaortic constriction)	↓ Plasma concentrations of noradrenaline, renin and BNP^h^	RGS-4 is an inhibitory factor of hypertrophy due to attenuation of G-protein mediating signalling	Liao et al. (2003)
						↓ Cardiac hypertrophy		
						↓ Interstitial and perivascular fibrosis		
						↑ Gene expression of RGS^i^-4		
						↓ Myocardial dysfunction and pulmonary congestion		
		CPA^j^ (selective)				↓ Cardiac hypertrophy	These effects were abolished by DPCPX^k^. Studies with CPA and DPCPX to evaluate the role of A_1_AR on cardiac fibrosis were not performed	
						↓ Myocardial dysfunction		
		CPA (selective)			C57BL/6 mice (phenylephrine)	Upregulation of A_1_AR	CPA did not attenuate AngII^l^ or IGF^m^-1-induced cardiac hypertrophy	Puhl et al. (2016)
						↓ Cardiac remodelling		
						↓ Oxidative stress		
		CAP^n^ (partial)			Dogs (microembolization-induced HF^o^)	↓ Cardiac remodelling		Sabbah et al. (2013a)
						↑ LV function		
						↑ Capillary density and oxygen diffusion distance		
						↓ Rate of MPTP^p^ opening		
						Normalized SERCA^q^-2a activity and expression of UCP^r^-2 and -3 and GLUT^s^-1 and -4		
		Neladenoson (selective partial)			11 patients with HFrEF^t^ treated on *ß*-blockers therapy	No 2nd or 3rd degree atrioventricular block occurred on 48-h Holter monitoring	Significant early changes in cardiac function were not detected.	Voors et al. (2017)
					31 patients with HFrEF^u^ on *ß*-blockers therapy	No 2nd or 3rd degree atrioventricular -block occurred on 48-h Holter monitoring.	No significant change in renal function and neurological side effects were detected	
						No effects were observed in heart rate and blood pressure		
		Neladenoson (selective partial)			261 patients with HFpEF	No significant dose-response regarding to the change in exercise capacity.		Shah et al. (2019)
						No significant improvement in markers of cardiac structure and function related to HFpEF progression.		
						Dose-dependent declines in renal function and heart rate were observed		
	A_2A_AR			Overexpressed A_2A_AR	Mice (transverse aortic constriction)	Attenuation of ↓ cardiac function	GATA-4 controls several genes that are upregulated in cardiac hypertrophy, including *ß*-MHC, cardiac troponin-C, atrial natriuretic factor, NCX^x^ and A_1_AR	Hamad et al. (2012)
						↓ Inflammatory factors genes expression		
						↓ *ß*-MHC^v^, ANP^w^, and GATA-4 gene expression		
						↑ Intracellular calcium homeostasis		
		CGS21680 (selective)			C57BJ/6J mice (DOCA^y^- salt)	↓ Cardiac inflammation, fibrosis, hypertrophy, and dysfunction	iBAT surgery depletion induces significantly cardiac remodelling refractory to the cardioprotective function of CGS21680	Zhou et al. (2020)
						↑ iBAT^z^-derived FGF21^i^		
	A_3_AR			A_3_AR KO	Mice (transaortic constriction)	↓ Cardiac remodelling	Attenuation of LV hypertrophy and dysfunction by reducing activation of the MAPK^ii^ and PI3K-Aκt^iii^ signalling pathways	Lu et al. (2008)
						↓ Oxidative stress		
						↓ANP and LV dysfunction		
Pulmonary hypertension	A_2A_AR			A_2A_AR KO	Mice (hypoxia)	↑ RVSP^iv^ and Fulton index	RhoA and ROCK are signalling pathways related to pulmonary vascular remodelling and PAH^v^ generation	Shang et al. (2015)
						↑ Thickness in pulmonary resistance vessels		
						↑ RhoA and ROCK expression		
		LASSBio-1359 (selective)			Wistar rats (MCT^vi^-IPHM^vii^)	↑ PA^viii^ vasorelaxation and flow		Alencar et al. (2013)
						↓ Pulmonary vascular remodelling		
						↓ RV^ix^ remodelling and RVSP		
						↑ LV stroke volume and cardiac output		
		LASSBio-1386 (selective)			Wistar rats (MCT-IPHM)	↑ Exercise tolerance	A_2A_AR agonism attenuates RV dysfunction indirectly by counteracting structural and functional changes in pulmonary vasculature	Alencar et al. (2014)
						↑ PA vasorelaxation and flow		
						↓ Pulmonary vascular remodelling		
						↓ RV remodelling and RVSP		
						↑ LV stroke volume and cardiac output		
						↑ eNOSx^x^ and A_2A_AR expression in lungs		
						↑ A_2A_AR and SERCA2a expression in RV		
						↑ Ca^2+^-ATPase activity in RV		
	A_2B_AR				13 COPD^xii^ with or without PH^xiii^patients	↑ Pulmonary vascular remodelling in COPD-PH patients		Karmouty-Quintana et al. (2013)
						↑ A_2B_AR gene and protein expression		

LV^a^, Left ventricle; AR^b^, Adenosine receptor; KO^c^, Knockout; AMP^d^, 5′-adenosine monophosphate; mTOR^e^, Mechanistic target of rapamycin; ADK^f^, Adenosine kinase; CADO^g^, 2-chloroadenosine; BNP^h^, B-type natriuretic peptide; RGS^i^, Regulator of G protein signalling; CPA^j^, N6-cyclopentyladenosine; DPCPX^k^, 8-Cyclopentyl-1,3-dipropylxanthine; AngII^l^, Angiotensin II; IGF^m^, Insulin growth factor; CAP^n^, Capadesonon; HF^o^, Heart failure; MPTP^p^, Membrane permeability pores; SERCA^q^, sarco/endoplasmic reticulum Ca2+-ATPase; UCP^r^, Uncoupling protein; GLUT^s^, Glucose transporter type; HFrEF^t^, Heart failure with reduced ejection fraction; HFpEF^u^, Heart failure with preserved ejection fraction; *ß*-MHC^v^, myosin heavy chain-beta; ANP^w^, Atrial natriuretic peptide; NCX^x^, Sodium-calcium exchanger; DOCA^y^, Deoxycorticosterone acetate; iBAT^z^, interscapular brown adipose tissue;FGF21^z^, Fibroblast growth factor 21; MAPK^ii^, mitogen-activated protein kinase; PI3K-Aκt^i^, phosphoinositol-3 kinase-protein kinase B; RVSP^iv^, Right ventricle systolic pressure; PAH^v^, Pulmonary arterial hypertension; MCT^vi^, Monocrotaline; IPHM^vii^, Induced pulmonary hypertension model; PA^viii^, Pulmonary artery; RV^ix^, Right ventricle; eNOS^x^, Endothelial nitric oxide synthase; PDE5i^xi^, Phosphodiesterase type 5 inhibitor; COPD^xii^, Chronic obstructive pulmonary disease; PH ^xiii^, Pulmonary hypertension.

### Microvascular Dysfunction

The current trend is that coronary microvascular dysfunction may be a cornerstone of the complex molecular pathways driving the clinical manifestations of HFpEF (D’Amario et al., 2019). Coronary microvascular dysfunction is inevitably associated with a compromised vasodilatory response and consequently to the existence of myocardial focal ischemic lesions contributing to diastolic dysfunction of the left ventricle (LV) (Tschope et al., 2005). This feature is further compromised by systemic comorbidities and local inflammatory states ending up into structural and functional abnormalities of the heart and blood vessels (D’Amario et al., 2019). Diastolic dysfunction, the hallmark of HFpEF, is related to coronary microvascular dysfunction, independently from the presence of coronary artery disease (D’Amario et al., 2019).

Experimental models of diabetes, obesity and metabolic syndromes predict that systemic inflammation unbalances the equilibrium between endothelium-derived relaxing factors (e.g., NO) and endothelium-derived vasoconstrictors (e.g., endothelin-1), thus increasing stiffness and vasoconstriction of large vessels at rest (Paulus and Tschope, 2013). Endothelial activation favors the expression of adhesion molecules, trans-endothelial migration of circulating leucocytes, and release of pro-inflammatory cytokines and ROS, thus contributing to perpetuate local inflammation (Westermann et al., 2011). Moreover, vascular inflammation decreases NO bioavailability (Griendling et al., 2000; Westermann et al., 2011) resulting in downstream reduction of soluble guanylate cyclase activity, low levels of cyclic GMP and decreased activation of protein kinase G, which is normally associated with titin hypophosphorylation, increased rigidity and myocardial diastolic dysfunction (Paulus and Tschope, 2013).

The vascular endothelium is a key player linking metabolic disorders and HFpEF. Excessive production of ROS by endothelial Nox2 activation has been associated with other cardiovascular diseases, such as hypertension and atherosclerosis, which are common comorbidities of HFpEF (Landmesser et al., 2002). *In vitro* blockage of the A_2A_AR effectively inhibits ROS production by endothelial Nox2 and attenuates angiotensin II (AngII)-induced oxidative stress and endothelial dysfunction. This occurs through inhibition of MAPK activation and, consequently, by reducing p47phox phosphorylation and binding to Nox2 (Thakur et al., 2010). Taking together, these findings suggest a potential role for A_2A_AR antagonism to counteract AngII-induced Nox2 activation in endothelial cells (Thakur et al., 2010). However, contradictory results urge depending on the vascular territory considered; the A_2A_AR inactivation displays a protective role in reducing oxidative damage in neurodegenerative diseases (Pugliese et al., 2009) and in the heart (Ribe et al., 2008), whereas its inactivation increased tracheal ROS production from Nox2 (Nadeem et al., 2009).

As a matter of fact, the A_2A_AR activation is reported to play a major role in endothelium homeostasis, culminating in increased endothelial production of NO, and subsequent vasodilation in most vascular beds (Figure 3) (Li et al., 1998). Nevertheless, it has been shown that A_2A_AR inactivation preserved the endothelium-dependent vasodilation to acetylcholine, while reducing ROS production; this might be explained by the very low Nox2 expression in vascular smooth muscle cells, the predominant cellular component in the vessel wall (Thakur et al., 2010). Even though the A_2A_AR is mainly expressed in endothelial cells, its activation operates both endothelium-dependent and -independent relaxation of blood vessels. Controversy still exists on the receptor subtype (A_2A_AR and/or A_2B_AR) predominating on endothelial cells (Chiang et al., 1994; Iwamoto et al., 1994; Li et al., 1998). Endothelial dysfunction in small penile vessels underlying erectile dysfunction may be taken as an early surrogate of cardiovascular disease severity (Thompson et al., 2005). In this context, adenosine regulates smooth muscle tone in human corpora cavernosa through the activation of high-affinity A_2A_AR and low-affinity A_2B_AR located on smooth muscle fibers and endothelial cells, respectively, (Faria et al., 2006). Interestingly, corpora cavernosa from men with vasculogenic impotence is partially resistant to adenosine relaxation due to endothelial A_2B_AR dysfunction, while keeping almost unaltered relaxation of cavernosal vessels via A_2A_AR on the smooth muscle layer (Faria et al., 2006). Insufficient adenosine formation linked to deficits in CD73 enzymatic activity has been associated with age-dependent endothelial dysfunction and NO production deficits in mice (Mierzejewska et al., 2019), which may strengthen the production of the pro-inflammatory cytokine, IL-6, and endothelial adhesion molecules, like intercellular adhesion molecule-1 and vascular cell adhesion molecule-1 (VCAM-1) (Mierzejewska et al., 2019).

Another hallmark of coronary microvascular dysfunction in patients with HFpEF is coronary microvascular rarefaction (Mohammed et al., 2015). Thus, adenosine-induced angiogenesis via A_2A_AR, A_2B_AR, and A_3_AR activation may be relevant in this scenario (Hinze et al., 2012; Ahmad et al., 2013; Du et al., 2015). In addition, it has been reported that activation of the A_1_AR subtype increases capillary density and oxygen diffusion distance in dogs submitted to transverse aortic constriction (Table 1) (Sabbah et al., 2013a). Interestingly, increased intracellular adenosine levels in human endothelial cells lacking ADK lead to hypomethylation in DNA promotor regions of pro-angiogenic genes (Xu et al., 2017b). Epigenetic upregulation of pro-angiogenic genes favor endothelial proliferation *in vitro* and ischemia induced-angiogenesis *in vivo* (Xu et al., 2017b).

Besides its antithrombotic properties, the P2Y_12_R antagonist, ticagrelor, has been increasingly used in cardiometabolic diseases like DM, most probably because of its pleiotropic/anti-inflammatory effects, which at least some of them are shared with adenosine (Wernly et al., 2021). Interestingly, ticagrelor restores defective ATP release and inhibits adenosine uptake by red blood cells directly impacting the actions of these two purines (or its metabolites) on immunocytes and endothelial cells (Wernly et al., 2021), which might be useful to counteract the development of atherosclerosis and related metabolic syndrome. In this regard, adenosine receptors (namely the A_2A_AR subtype) counteract the development of atherosclerosis by providing endothelial/vascular protection. This effect is attributed to the nucleoside anti-inflammatory properties, as well as to its ability to modulate the cholesterol transport in macrophages and the hepatic metabolism of lipids (reviewed in (Koupenova et al., 2012)). Consistent with these findings, the THEMIS-PCI (The Effect of Ticagrelor on Health Outcomes in Diabetes Mellitus Patients-Percutaneous Coronary Intervention) sub-study revealed a reduction of major adverse cardiac events in diabetic patients submitted to percutaneous coronary intervention and treated with ticagrelor plus aspirin compared to aspirin monotherapy (Bhatt et al., 2019). A more comprehensive understanding about the mechanisms underlying ticagrelor-induced endothelial dysfunction improvement is needed to support indication of this drug for cardiometabolic diseases (Wernly et al., 2021).

### Interstitial Fibrosis and Loss of Myocardial Compliance

Structural abnormalities in HFpEF involve cardiomyocyte hypertrophy and interstitial fibrosis (Paulus and Tschope, 2013; Lam et al., 2018; Simmonds et al., 2020). Under pathological stressful conditions, unopposed actions of pro-fibrotic and pro-hypertrophic paracrine and neurohormonal signals further aggravate cardiac chambers stiffness and impaired relaxation. Diastolic dysfunction and inherent increased LV filling pressure foster secondary left atria remodelling and dysfunction, further aggravating HFpEF symptoms and long-term prognosis (Paulus and Tschope, 2013; Lam et al., 2018; Simmonds et al., 2020). Cardiac fibroblasts are the predominant interstitial cell type in the adult mammalian heart (Souders et al., 2009). Thus, it is not surprising that cardiac fibrosis and associated decrease in myocardial compliance represents a common pathological hallmark in the failing heart (Paulus and Tschope, 2013; Simmonds et al., 2020).

Among all adenosine receptors, A_2A_AR emerges by its cAMP-dependent anti-fibrotic action (Sassi et al., 2014). Separating the contribution of both A_2A_AR and A_2B_AR to fibrotic processes has been difficult due to the lack of selective drugs used in the past (Vecchio et al., 2017). As aforementioned, the cardiac anti-inflammatory/anti-fibrotic role of A_2A_AR has been translated into disease models of HFpEF (Hamad et al., 2012; Quast et al., 2017) and ischemia/reperfusion (Jordan et al., 1997; Yang et al., 2006; Rork et al., 2008). Activation of the A_2A_AR decreased the expression of fibrosis-related genes and cardiac fibrosis, while improving cardiac remodelling and function, in the deoxycorticosterone acetate-salt induced hypertensive mice model of cardiac remodelling (Table 1) (Zhou et al., 2020). One may, therefore, hypothesize that the anti-fibrotic effect of adenosine via A_2A_AR activation is operated either, directly, by inhibiting growth and/or differentiation of cardiac fibroblast, or, indirectly, through nucleoside-induced immunosuppressive effects, thereby preventing pro-fibrotic cells chemotaxis and the release of inflammatory mediators. In addition to these possibilities, adenosine deamination to inosine by third-party ADA cell providers (e.g., inflammatory cells) may also play a role in the anti-fibrotic effect of adenosine, as we recently demonstrated in human subcutaneous fibroblasts (Herman-de-Sousa et al., 2020). Thus, clarification of the mechanism and receptor involved in the anti-fibrotic role of adenosine in myocardial fibrosis is warranted given to the fact it may exert pro-fibrotic effects in other organs, such as the liver (Chan et al., 2006) and the skin (Herman-de-Sousa et al., 2020), while the opposite is verified with the A_2B_AR which inhibits fibrosis in the heart (see below) and promotes fibrosis of the lung (reviewed in (Bessa-Goncalves et al., 2018)).

The low-affinity A_2B_AR is the most abundant receptor in rat cardiac fibroblasts (Epperson et al., 2009). Therefore, it is not surprising that the anti-fibrotic effect of the A_2B_AR is relatively consensual, at least *in vitro* (Chen et al., 2004), while questions may arise about the endogenous amounts of the nucleoside required to activate this low-affinity receptor under both normal and pathological conditions. Nevertheless, it has been shown that intracellular cAMP accumulation prevents TGF-β-, AngII-, and endothelin-1-induced collagen synthesis and *a*-smooth muscle actin expression by activated cardiac myofibroblasts via EPAC and PI3K dependent pathways (Delaunay et al., 2019). Likewise, fibroblast proliferation, collagen synthesis, *a*-smooth muscle actin expression and myofibroblast differentiation were all attenuated by cAMP-coupled A_2B_AR activation, thus counteracting pro-fibrotic stimuli induced by endothelin-1 and AngII (Figure 3) (Delaunay et al., 2019) via a mechanism involving cAMP and EPAC downstream pathways (Phosri et al., 2017; Phosri et al., 2018). The cAMP/EPAC/PI3K/Akt signalling pathway was also involved in A_2B_AR-mediated suppression of fibroblasts growth and *a*-smooth muscle actin-expressing myofibroblast differentiation induced by endothelin-1 (Phosri et al., 2017). In a canine *in vivo* model of HF it was found that the EPAC1 content in fibroblasts of left atria exhibiting fibrotic remodelling was significantly downregulated (Surinkaew et al., 2019). Taking together, data suggest A_2B_AR coupling to the cAMP/EPAC pathway may provide potential targets to prevent/overcome cardiac fibrosis that may be required to rescue myocardial compliance.

Even though A_2B_AR agonists exhibit cardiac anti-fibrotic effects *in vitro*, these findings were not consistently translated into *in vivo* data, most probably due to the dual action of this receptor in cardiac inflammation (Vecchio et al., 2017). Available data suggests that A_2B_AR activation before ischemia followed by reperfusion may have a cardioprotective effect, most likely because of the inhibition of pro-inflammatory/pro-fibrotic cytokines release via PI3K/Akt pathway (Eckle et al., 2007b; Tian et al., 2015b; Ni et al., 2018). Moreover, a more complex interplay between A_1_AR and A_2B_AR subtypes is also possible, given to the fact that apparently the A_1_AR-operated cardiac protection relies on A_2B_AR overexpression by a PKC-dependent pathway during ischemia and preconditioning (Kuno et al., 2007). Nevertheless, in contrast to the A_2A_AR, there is no consensus about the putative beneficial roles of the A_2B_AR on the remodeling phase after myocardial ischemia (Sun and Huang, 2016), with studies demonstrating a protective role of either A_2B_AR blockage (Toldo et al., 2012; Zhang et al., 2014) or activation (Wakeno et al., 2006). Blockage of A_2B_AR after MI occurrence seems to attenuate PKC-δ/p38-MAPK-mediated secretion of pro-inflammatory and pro-fibrotic mediators, like TGF-β, TNF-α, and IL-6 (Feng et al., 2010; Toldo et al., 2012; Zhang et al., 2014). On the other hand, deletion of A_2B_AR did not change fibrosis in the remodelling phase after myocardial ischemia, probably due to opposing pro- and anti-inflammatory stimuli (Alter et al., 2019). In fact, the injured heart depends mostly on the low-affinity A_2B_AR probably due to functional inactivation of the A_2A_AR through dimerization (Hinz et al., 2018). It is worth noting that A_2B_AR may couple to both G_s_ and G_q_ proteins, as well as to other G proteins; its preferential coupling to a certain G protein dependents on multiple factors, including the cell type, receptor expression levels, and chemical nature of the agonist - “biased receptor” function (reviewed in (Bessa-Goncalves et al., 2018)). Thus, clarification of the precise mechanisms involved in pro-vs. anti-fibrotic effects of the A_2B_AR in the heart is still needed to correctly infer any benefit derived from the manipulation of the activity of this receptor in patients with HFpEF.

### Cellular Structural Changes

Overload pressure, oxidative stress and tissue injury, as well as the influence of neurohormonal and inflammatory mediators, like AngII, endothelin-1, catecholamines, growth factors and TNF-α, promote ventricular hypertrophy (reviewed in (Nakamura and Sadoshima, 2018)). While cardiac hypertrophy may be considered a physiologically adaptive response to external insults, over time it becomes pathological and facilitates progression to HF (Nakamura and Sadoshima, 2018; Simmonds et al., 2020).

The myocardial adenosine content highly increases in hypertrophied hearts (Funaya et al., 1997; Asakura et al., 2007). The nucleoside counteracts the activity of many neurohumoral factors involved in cardiac hypertrophy, namely endothelin-1 (Stowe et al., 1997), renin-angiotensin-aldosterone system (Taddei et al., 1992) and TNF-α (Wagner et al., 1998), and noradrenaline release from sympathetic nerves (Burgdorf et al., 2001). Considering these features, it was hypothesized that adenosine may exert an anti-hypertrophic action and, in this sense, might counteract cardiac dysfunction (Liao et al., 2003). Indeed, inhibition of adenosine uptake with dipyridamole increased myocardial adenosine levels, which significantly ameliorated LV filling and pulmonary congestion, while attenuating *ß*-adrenergic dysfunction in rats with pressure overload cardiac hypertrophy (Table 1) (Chung et al., 1998). Conversely, inhibition of adenosine formation from released adenine nucleotides in CD73 deficient mice promoted myocardial hypertrophy, LV dilation and LV dysfunction (Table 1) after aortic constriction, but not in healthy animals (Xu et al., 2008).

The A_1_AR seems to play a chief role in the putative anti-hypertrophic action of adenosine in the heart (Liao et al., 2003; Sabbah et al., 2013a; Puhl et al., 2016). The enzymatically stable A_1_AR agonists, 2-chloroadenosine (CADO) and N^6^-cyclopentyladenosine (CPA), significantly reduced the plasma concentrations of noradrenaline, renin, and brain natriuretic peptic in a mice model of transverse aortic constriction-induced HFpEF (Table 1) (Liao et al., 2003). When used *in vivo*, these drugs also effectively attenuated cardiomyocytes hypertrophy and fibrosis in interstitial and perivascular spaces through A_1_AR activation, resulting in improved cardiac function and reduced pulmonary congestion (Liao et al., 2003). Both CADO and CPA attenuated protein synthesis induced by activation of G_q_-protein-coupled receptors for AngII, endothelin-1, and noradrenaline, as well as the PKA-mediated hypertrophic actions of isoproterenol in neonatal rat cardiomyocytes (Liao et al., 2003). Interestingly, *in vivo* stimulation of α1-adrenoceptor with phenylephrine upregulated A_1_ARs expression. Activation of overexpressed A_1_AR with CPA counteracted the hypertrophic phenotype caused by phenylephrine, but the same was not verified concerning cardiac hypertrophy/fibrosis caused by AngII and IGF1 (Table 1) (Puhl et al., 2016). These findings suggest that different signalling pathways operate maladaptive cardiac responses to distinct neurohumoral agents, namely AngII and phenylephrine (Puhl et al., 2016).

Adenosine counteraction of cAMP accumulation induced by *ß*-adrenoceptors stimulation (anti-adrenergic effect) has been proposed to explain the anti-hypertrophic effect of the nucleoside (Figure 3) (Meyer et al., 2001). Besides this, adenosine by positively modulating G protein signaling 4 may counteract Gq protein-induced hypertrophy, which may contribute to the anti-hypertrophic effect of the nucleoside against α1-adrenoceptor agonists (Liao et al., 2003). A_1_AR-receptor-mediated interference on intracellular Ca^2+^ handling may also play a role against myocytes hypertrophy probably by downregulating the calcineurin pathway (Meyer et al., 2001). In this context, sustained *ß*-adrenergic activation increases interstitial fibrosis and myocyte hypertrophy, two features of myocardial remodelling and HF (Prijic and Buchhorn, 2014). Upon *ß*-adrenergic stimulation, cardiomyocytes let intracellular cAMP flow to the extracellular space via the ATP-binding cassette sub-family C member 4 transporter where it may act as an important paracrine cardioprotective signalling molecule. Once in the extracellular space, cAMP may be enzymatically converted to AMP by ecto-phosphodiesterases, which will then be dephosphorylated to adenosine by ecto-5′nucleotidase/CD73. In this sense, one may hypothesize that extracellular adenosine yields may partially counteract myocardial hypertrophy due to *ß*-adrenoceptors overactivation through activation of A_1_AR negatively coupled to the AC while delivering anti-fibrotic signals to cardiac fibroblasts through A_2A_AR activation (Figure 3) (Sassi et al., 2014). Thus, the anti-adrenergic effect of adenosine may serve as an endogenous local *ß*-blocker by limiting intracellular cAMP formation with inherent beneficial cardiac effects. In line with this view, capadesonon, a partial A_1_AR agonist, decreased myocardial hypertrophy and fibrosis, while increasing capillary density and oxygen supply, which significantly improved LV function in dogs with advanced HF (Table 1) (Sabbah et al., 2013a). All together these pieces of evidence suggest that A_1_AR may be a potential target to prevent transition from compensated myocardial hypertrophy to irreversible HF.

In addition to the A_1_AR-mediated anti-hypertrophic effect of adenosine, A_2A_AR overexpression or agonist activation attenuated cardiomyocyte hypertrophy (Hamad et al., 2012; Zhou et al., 2020) and decreased hypertrophy-associated genes encoding *ß*-myosin heavy chain and atrial natriuretic peptide along with their transcription activator protein GATA-4 in mice with pressure-induced HF secondary to transverse aortic constriction (Hamad et al., 2012). These cardioprotective A_2A_AR-mediated effects are associated with attenuation of the inflammatory response via MAPK- and PKC-operated pathways independently of PKA activation (Hamad et al., 2012).

Considering the cardioprotective role of adenosine against chronic cardiac pressure overload, unexpected results were obtained using A_3_AR knockout (KO) mice. These animals exhibited partial resistance to transverse aortic constriction-induced LV hypertrophy, fibrosis, and oxidative stress (Table 1) (Lu et al., 2008). Hemodynamically, A_3_AR KO mice exhibited higher ejection fractions and smaller LV end-systolic diameters; the plasma levels of the atrial natriuretic peptide in these animals were also lower compared to wild type controls confirming less cardiac hypertrophy. Authors interpreted these findings by suggesting that A_3_AR activation might potentiate the inflammatory response triggered by cardiac pressure overload, thus favoring LV hypertrophy and cardiac dysfunction (Lu et al., 2008). In this context, it was interesting to verify that selective blockage of A_3_AR with MRS1911 potentiated the anti-hypertrophic effect of CADO in cardiomyocytes treated with phenylephrine, strengthening the idea that activation of the A_3_AR might have a deleterious effect in cardiac remodelling following chronic pressure overload (Lu et al., 2008). The effects obtained with the A_3_AR antagonist were remarkably similar to those verified upon reducing the activation of MAPK and PI3K-Aκt signalling pathways (Lu et al., 2008), which are often activated in response to oxidative stress and inflammation (Nakamura and Sadoshima, 2018). Confirmation of the protective role of A_3_AR blockage on the inflammatory component attenuating pressure overload cardiac remodelling requires the use of more potent and selective drugs. It is also worth noting that the adenosine deamination metabolite, inosine, may exert opposite effects to adenosine in the heart via A_3_AR receptors, which activation is strengthened when tissues become infiltrated by ADA-bearing inflammatory cells (Herman-de-Sousa et al., 2020). This concept raises new lines of research using multi-target drugs designed to increase local adenosine levels while decreasing inosine formation (e.g., ADA inhibitors with blocking properties of adenosine cellular uptake, like the lymphocytotoxic drug 2′-deoxycoformycin or pentostatin) applied in combination with enzymatically stable adenosine receptor agonists and/or antagonists (Herman-de-Sousa et al., 2020).

There is evidence that adenosine may also exert cardioprotective actions in the heart via non-receptor-mediated mechanisms. In support of a role for intracellular adenosine metabolism in regulating hypertrophy, it has been demonstrated that ADK inhibitors, such as iodotubercidin and ABT-702, completely reversed the anti-hypertrophic actions of adenosine and its enzymatically stable analogue, CADO, in phenylephrine-induced hypertrophic neonatal rat cardiomyocytes (Fassett et al., 2011); data identified ADK as an important mediator of adenosine attenuation of cardiomyocyte hypertrophy acting, at least in part, through inhibition of Raf signalling to mTOR/p70S6k. The role of ADK in the anti-hypertrophic effect of adenosine has also been demonstrated *in vivo* in a KO mice model of cardiac pressure overload induced by transverse aortic constriction (Table 1) (Fassett et al., 2019). ADK enzymatic activity dampened cardiac growth signaling (by mTOR complex 1 and Erk), as well as the excessive microtubule stabilization/detyrosination (Fassett et al., 2019). Overall, these data points towards a novel ADK-dependent adenosine receptor-independent mechanism to protect against adverse hypertrophy remodelling and excessive cardiomyocyte microtubule stabilization.

### Cardiometabolic Dysfunction

Mechanisms underlying impaired myocardial energetics in HF can be divided into three subgroups: 1) abnormal mitochondrial structure and function; 2) change in substrate utilization; and 3) intracellular Ca^2+^ overload (Birkenfeld et al., 2019).

The failing heart is characterized by abnormal mitochondrial structure and function, including hyperplasia and reduced organelle size, inadequate organelle respiration, reduced mitochondrial membrane potential, and opening membrane permeability pores. Therefore, ATP synthesis is reduced because of impaired electron transport chain and excessive ROS generation by mitochondria (Birkenfeld et al., 2019). Association between A_1_AR activity and mitochondrial dysfunction has been observed in HF (Figure 3) (Sabbah et al., 2013a). Treatment with the partial A_1_AR agonist, capadesonon, caused a near normalization of mitochondrial dysfunction in animal models of HF (Table 1) (Sabbah et al., 2013a). Capadesonon decreased the opening rate of mitochondrial permeability transition pores resulting in reduced apoptosis and normalization of citrate synthase expression, used as a marker of unaltered mitochondrial function (Sabbah et al., 2013a). Likewise, the A_1_AR agonist, 2-choloro-N^6^-cyclopentyladenosine, reduced the permeability of mitochondrial transition pores in rat cardiomyocytes submitted to hypoxia through a mechanism involving PKC activation, stabilization of mitochondrial membrane potential and inhibition of ROS production, culminating with the opening of K_ATP_ mitochondrial channels (Xiang et al., 2010b). The low-affinity A_2B_AR has also been associated with the reduction of the production of mitochondrial ROS via a mechanism involving PI3K, Erk, and NOS in adult rabbit cardiomyocytes (Yang et al., 2011).

Due to abnormal mitochondrial structure and function with consequent impairment of the electron transport chain, there is a switch in the cellular energetics in the failing heart from a preferential metabolism of fatty acids to glucose. This shift in the cellular energetics to anaerobiosis in order to spare oxygen consumption is potentiated by A_1_AR activation. In fact, this receptor has been shown to restore the plasma membrane expression of glucose transporters 1 and four to normal levels (Table 1) (Sabbah et al., 2013a) and to decrease the levels of free fatty acids (Staehr et al., 2013; Cui et al., 2021).

Intracellular Ca^2+^ overload in HF causes abnormal function of the sarco/endoplasmic reticulum Ca^2+^-ATPase (SERCA)2a, which normally pumps Ca^2+^ from the cytosol into the sarcoplasmic reticulum during diastole; as a consequence, active muscle relaxation is impaired and ventricular cardiomyocyte stiffness becomes an issue (Lam et al., 2018). Activation of A_1_AR with capadesonon ameliorated SERCA2a function, which by restoring the buffering Ca^2+^ capacity of the sarcoplasmatic reticulum indirectly facilitates the diastolic relaxation of cardiomyocytes, while also restraining intracellular Ca^2+^ overload before the next contraction (Table 1) (Sabbah et al., 2013a). On the other hand, although the activation of the A_2A_AR increases intracellular Ca^2+^ transients during systolic activity, they rapidly subside during diastole in a mouse model of cardiac pressure overload due to transverse aortic constriction (Hamad et al., 2012), through a PKA dependent mechanism (Table 1) (Hamad et al., 2012).

## Important Comorbidities in HFpEF

### Diabetes Mellitus and Metabolic Syndrome

*Diabetes mellitus* (DM) is frequently associated with the occurrence and severity of HF; roughly 30–40% of patients with HFpEF are also affected by this endocrinopathy (Campbell et al., 2012). According to the clinical trial RELAX (Phosphodiesterase-5 Inhibition to Improve Clinical Status and Exercise Capacity in Diastolic Heart Failure), diabetic HFpEF patients were younger, mostly males, and affected by other comorbidities, namely obesity, hypertension, renal dysfunction, pulmonary disease, and vascular disease (Lindman et al., 2014).

The role of adenosine in insulin resistance and obesity has been recently reviewed by our group (de Oliveira et al., 2020). Adenosine, via A_1_AR activation, suppresses lipolysis and plasma free fatty acids; increases lipogenesis and adipogenesis; it also regulates insulin sensitivity and glucose tolerance, indirectly interfering with myocardial substrate handling (Dhalla et al., 2007; Johansson et al., 2008). Moreover, it has been recently shown that adenosine, via ADA acting on RNA 1, may control appetite signaling and modulation along with limiting obesity and insulin resistance (Table 2) (Cui et al., 2021). ADA acting on RNA 1-deficient heterozygous mice fed with a high-fat diet (HFD) for 12 weeks exhibited a lean phenotype compared to wild-type controls which became obese within the same time period; the lean phenotype was associated with less severe dyslipidaemia and insulin resistance parameters, along with decreased food intake, decreased gastric ghrelin expression, and attenuated reduction of serum peptide YY levels (Cui et al., 2021). Altogether these findings suggest that ADA acting on RNA 1 may contribute to diet-induced obesity by modulating genes related to appetite control, such as ghrelin (“hunger hormone”) and peptide YY (“satiety hormone”) (Cui et al., 2021).

**TABLE 2:** Results of adenosine receptors agonism/antagonism in diabetes mellitus/obesity and chronic kidney disease. Different agonists/antagonists were used to assess its effects on HFpEF comorbidities. The grey area corresponds to studies carried out in human patients.

	Adenosine receptors	Adenosine receptor modulation			Model and species	Outcome	Comments	Refs
		Pharmacological tool						
		Agonist	Antagonist	Other				
Diabetes mellitus/Obesity				ADAR1^a^ deficiency	Mice (HFD^b^)	↓ Fat mass, dyslipidemia, and insulin resistance parameters	No difference was shown on fat mass in ADAR1 deficient mice under chow diet compared to HFD	Cui et al. (2021)
						↓ Food intake and gastric ghrelin		
						↑ Serum peptide YY		
	A_1_AR^c^	CPA^d^ (selective)			Wistar rats (STZ^e^ injection)	↓Plasma glucose, cholesterol, and triglyceride	Plasma glucose lowering effect mediated by endogenous insulin was negligible	Cheng et al. (2000)
						↑ Glucose uptake into peripheral tissues		
						↑ Uptake and glycogen synthesis		
	A_1_AR	GS-9667 (selective, partial)			55 non-obese and 23 overweight/obese patients	↓ Plasma FFA^f^	Partial A_1_AR agonism was aimed to reduce side effects	Staehr et al. (2013)
						Well-tolerated		
						No desensitization or rebound		
	A_1_ARA_2A_ARA_2B_AR (respectively)		DPCPX^g^SCH58261MRS1754 (all selective)		Wistar rats (HSu^h^ diet)	SCH58261 and MRS1754 decreased insulin sensitivity in control animals and improved insulin sensitivity in the skeletal muscle of HSu animals.DPCPX reverted the increase in total and visceral fat induced by HSu diet.	Adenosine exerts opposite effects on insulin sensitivity under control or resistant statesUnder context of insulin resistance, A_2_AR in the skeletal muscle, rather than on adipose tissue, is the main mediator of whole-body insulin sensitivity	Sacramento et al. (2020)
	A_2B_AR		MRS1754 (selective)	ADK^i^ deficiency	Mice (HFD)	↑Glucose tolerance and insulin sensitivity ↓ Metabolic syndrome ↑ Intracellular adenosine↑ NO^j^ production due to ↑ NOS3^k^ activity and modulation of NOS3 by A_2B_AR	Feeding mice with a HFD enhanced expression of endothelial ADK. A_2B_AR antagonism abolished NOS3 expression, but was not associated with increased phosphorylation of NOS3	Xu et al. (2019)
Renal disease				BT702 (ADK inhibition)	C57BL/6 mice (STZ injection)	↓ Hyperglycemia ↓ Albuminuria and markers of glomerular injury ↓ Renal oxidative stress and inflammatory parameters Restored glomerular permeability and filtration function	The reduced renal inflammation is consistent with the reduction in renal macrophage infiltration, NF-κB^l^ phosphorylation and MCP-1^m^ excretion The increased eNOS^n^ expression counteracts renal oxidative stress Attenuated glomerular damage is attributed to increased MAPK^o^ phosphorylation	Pye et al. (2014)
	A_1_AR	CCPA^p^ (selective)	DPCPX (selective)		C57BL/6 mice (renal I/R injury)	↑ Renal function ↓ inflammatory markers, necrosis, and apoptosis ↓ Renal function ↑ Inflammatory markers, necrosis, and apoptosis		Lee et al. (2004)
		CCPA (selective)			C57BL/6 mice (renal I/R injury)	↑ HSP27^q^ expression ↑ Renal function ↓ Pro-inflammatory cytokines ↓ Caspase 3 activity	HSP27 is an anti-apoptotic molecule as it is associated with an increased resistance to stressful conditions, such as oxidative stress and exposure to toxic drugs A_1_AR- mediated renal protection, anti-inflammatory and anti-apoptotic effects were removed by inhibition of HSP27	Xiong et al. (2018)
			DPCPX (selective)			↑ Natriuresis and diuresis ↓ HSP27 expression		
			KW-3902 (rolofylline)		32 patients with congestive HF^s^ and renal impairment	↑ GFR^t^ and renal plasma flow	The increased in GFR persisted more time than the predicted by pharmacokinetics	Dittrich et al. (2007)
			KW-3902 (rolofylline)		146 patients with volume overload and an estimated CrCl^r^ of 20–80 ml/min	↑ Urine output	A_1_AR antagonism enhances the response to loop diuretics	Givertz et al. (2007)
	A_2B_AR		MRS1754 (selective)		C75BL/6 mice (STZ injection)	↓ Albuminuria ↑ Renal function ↓ VEGF-A^u^ expression ↑ NO production ↓ Glomerulosclerosis	In diabetic mice kidney, there was a significant increase in VEGF-A and A_2B_AR gene expression	Patel and Thaker, (2014)
	A_2A_AR		ZM241385 (selective)	Spironolactone (mineralocorticoid receptor antagonist)	Sprague-Dawley rats (isoprenaline)	↑ A_2A_AR	ZM241385 exacerbated cardiorenal remodelling and dysfunction	Chen et al. (2019)
						↓ EndMT^v^ process		
						↓ Cardiac and renal fibrosis and dysfunction		
		CGS21680 (selective)			Sprague-Dawley rats (STZ injection)	↓ Glomerular hyperfiltration	A_2A_AR stimulation did not decrease filtrated fraction during NOS inhibition, demonstrating that A_2A_AR-mediated vasodilatation of efferent arterioles to be NO dependent	Persson et al. (2015b)
						↓ Albuminuria		
						↓ Immune cells infiltration		
						↓ Urine excretion of pro-inflammatory cytokines		
		CGS21680 (selective)			Sprague-Dawley rats (STZ injection)	↓ Proteinuria and glomerular damage	These results that A_2A_AR stimulation exerts renoprotective effects via an anti-inflammatory mechanism	Persson et al. (2015a)
						↓ TNF-α^w^ secretion and macrophages infiltration		
-		CADO^x^ (non selective)			SHR^y^-STZ rats	↓ Hyperglycaemia and glycosuria		Patinha et al. (2020)
						↓ Proteinuria		
						↓ Collagen deposition and oxidative stress in the renal glomeruli		
						↑ Immunoreactivity against A_2A_AR		
			DPSPX^z^ (non selective)			↑ Renal fibrosis↓ Immunoreactivity against A_3_AR	Treatment with DPSPX did not altered the global metabolic status and renal function.	
							The downregulation of A_3_AR may reflect a renoprotective mechanism	
	A_3_AR		LJ-6898 (selective)		C57BLKS/J-db/db	↓ Albuminuria and glomerular hypertrophy ↓ Renal fibrosis, inflammation, and oxidative stress ↓ Lipid accumulation ↑ PCG1α^z^ ↑ A_2A_AR expression	These effects were on the same range of losartan PCG1α is key regulator of mitochondrial biogenesis	Dorotea et al. (2018)

ADAR1^a^, ADA acting on RNA 1; HFD^b^, High fat diet; AR^c^, Adenosine receptor; CPA ^d^, N6-cyclopentyladenosine; STZ^e^, Streptozotocin; FFA^f^, Free fatty acid; DPCPX^g^, 8-Cyclopentyl-1,3-dipropylxanthine; HSu^h^, High-sucrose; ADK^i^, Adenosine kinase; NO^j^, Nitric oxide; NOS^k^, Nitric oxide synthase; NF-κB^l^, Nuclear factor-kappa B; MCP-1^m^, Monocyte chemoattractant protein-1; eNOS^n^, Endothelial nitric oxide synthase; MAPK^o^, Mitogen-activated protein kinases; CCPA^p^, 2-Chloro-N6-cyclopentyladenosine; HSP27 ^q^, Heat shock protein 27; CrCl^r^, Clearance of creatinine; HF^s^, Heart failure; GFR ^t^, Glomerular filtration rate; VEGF-A^u^, Vascular endothelial growth factor-A; EndMT^v^, Endothelial-to-mesenchymal transition; TNF-α^w^, Tumour necrosis factor-alpha; CADO^x^, 2-chloroadenosine; SHR^y^, Spontaneously hypertensive rat; DPSPX^z^, 1,3-dipropyl-8-sulfophenylxanthine.

In mice with streptozotocin-induced diabetes, activation of the A_1_AR with CPA decreases plasma glucose, cholesterol, and triglyceride levels, and improves glucose utilization in peripheral tissues by increasing its uptake and glycogen synthesis by target cells (Table 2) (Cheng et al., 2000). In this context, A_1_AR agonists have been proposed for the treatment of type 2 DM and obesity (see *Pros and Cons of Adenosine A*_*1*_ *Receptor Manipulation in HFpEF*).

Activation of the low-affinity A_2B_AR has been given special attention as a putative therapeutic target for obesity due to its ability to inhibit adipogenesis, increase lipolysis, improve insulin signaling and decrease white adipose tissue mass (Gharibi et al., 2012; Peleli et al., 2015). Activation of A_2A_AR increases lipolysis; this receptor is by far more expressed in the brown adipose tissue compared to the white adipose tissue, which might explain why it favors thermogenesis (de Oliveira et al., 2020). These findings, together with the anti-inflammatory potential of A_2A_AR activation, put this receptor in good position to counteract insulin resistance (de Oliveira et al., 2020). Previous results using the deoxycorticosterone acetate-salt induced hypertensive mice model showed that A_2A_AR activation promotes the release of fibroblast growth factor 21 by brown adipocytes, which presence seems to be crucial to combat hypertensive cardiac remodelling (Table 1) (Zhou et al., 2020). Confounding data, however, emerged from studies where the chronic consumption of caffeine reduced the risk of insulin resistance and type 2 DM (van Dam et al., 2006). It is worth noting that caffeine is the most widely psychoactive substance consumed worldwide and it acts mainly through the antagonism of adenosine receptors under moderate consumption of caffeine-containing beverages. A recent study carried out in Wistar rats of both gender fed either with a normalized chow or with a high-sucrose diet showed for the first time that chronic adenosine receptor blockage with caffeine favors insulin resistance in control animals, while restoring insulin sensitivity in animals treated with the high-sucrose diet (Table 1) (Sacramento et al., 2020). These authors also made clear that whole-body insulin sensitivity is under the control of both A_2A_AR and/or A_2B_AR given to the fact that selective blockage of each of these receptors rescued insulin sensitivity in skeletal muscles, both in males and females. Concerning the adipose tissue, chronic A_1_AR antagonism decreased fat accumulation, whereas the opposite was observed upon blockage of A_2A_AR and/or A_2B_AR; in this respect, gender differences were found with A_1_AR blockage being more effective in females, while antagonism of the A_2_AR was more powerful in males. Overall, these findings indicate that the effect of adenosine against insulin resistance via A_2A_AR and/or A_2B_AR is more prominent in skeletal muscles rather than in the adipose tissue where the A_1_AR seems to predominate (Sacramento et al., 2020).

Accumulating evidence suggest a role for adenosine in endothelial homeostasis maintenance. Due to its high affinity for the nucleoside, ADK is a key intracellular enzyme that is responsible for maintaining low intracellular adenosine levels while consistently driving the nucleoside to the purine nucleotides salvage pathway (Boison, 2013). Deletion of ADK, leading to increased intracellular adenosine accumulation and subsequent outflow of the nucleoside from cells, was found to increase *ß*-cell replication, thereby protecting against HFD-induced glucose intolerance (Navarro et al., 2017). Endothelial ADK was significantly upregulated in mice submitted to HFD; conversely, endothelial-specific ADK deficiency protected mice from HFD-induced insulin resistance and metabolic syndrome. This may be due to increased adenosine translocation to the extracellular milieu where it can activate plasma-membrane bound receptors, including endothelial A_2B_AR, which downstream signals contribute to phosphorylate NOS 3 fostering NO production and NO-related protective anti-inflammatory effects due to vasodilation and angiogenesis (Table 2) (Xu et al., 2019). The anti-inflammatory potential of ADK inhibition is also associated with reduced leucocyte adhesion to the endothelium, further contributing to regulation of glucose homeostasis and insulin sensitivity (Xu et al., 2017a).

### Renal Dysfunction

Due to population aging and incorrect lifestyle habits the prevalence of HFpEF and chronic kidney disease is dramatically increasing (Fang, 2016; Lofman et al., 2017). HFpEF has been considered a “vicious cycle” disorder of kidney and cardiovascular function disequilibrium, as either HFpEF or chronic kidney disease may accelerate the pathological progression of each other. Both disease conditions have not only overlapping comorbidities, including association with hypertension, diabetes and obesity, but also share common underlying features, like hypervolemia, systemic inflammation, and endothelial dysfunction (Fang, 2016).

Adenosine has been reported to reduce urinary protein excretion (Patinha et al., 2014), an earlier marker of underlying kidney injury causing renal dysfunction (Katz et al., 2014), by acting at the kidney level (reviewed in (Pandey et al., 2021)). Specifically, adenosine controls renin release, glomerular filtration rate, tubuloglomerular feedback mechanism and vascular tone through all four P1 receptor subtypes.

The A_1_AR plays an important role in renal physiology, as it controls afferent arteriole vasoconstriction, reabsorption of sodium in the proximal tubule and the tubuloglomerular feedback. Through inhibition of renin release from justaglomerular cells along with renal arteriole vasodilation, adenosine (via A_1_AR) reduces renal blood flow lowering the glomerular filtration pressure (Pandey et al., 2021), which is protective against kidney damage caused by metabolic diseases, such as diabetes and hypertension, often complicating HFpEF (Lofman et al., 2017; Pandey et al., 2021). The salutary effects of sodium-glucose cotransporter-2 (SGLT2) inhibitors, like empagliflozin, on diabetes-related hyperglycaemia, obesity and hypertension may be at least in part mediated by the reduction of glomerular hyperfiltration via activation of A_1_AR receptors and TGF-mediated afferent arteriolar vasoconstriction, independently of their ability to lower the plasma glucose levels (Kidokoro et al., 2019). The use of SGLT2 inhibitors provides a new perspective to the cardio- and reno-protective role of A_1_AR activation, as it decreases the intraglomerular pressure and prevents hyperfiltration, which may counteract chronic kidney disease progression and impaired renal function, indirectly ameliorating the cardiovascular outcome (Kidokoro et al., 2019). Moreover, renal activation of A_1_AR reduces the metabolic demand, tubular cell apoptosis and inflammatory reactions in mice models of ischemia/reperfusion (Table 2) (Lee et al., 2004; Xiong et al., 2018), thus strengthening the reno-protective effects of A_1_AR agonists.

The ability of A_1_AR antagonists to induce natriuresis without compromising the glomerular filtration rate suggests that this approach may be beneficial to control volume overload disorders, such as HF (Gottlieb et al., 2002), yet controversy still exists in the literature regarding this issue (see details in *Pros and Cons of Adenosine A*_*1*_ *Receptor Manipulation in HFpEF*). Interestingly, long-term dual blockage of A_1_AR/A_2B_AR with the orally-available drug tonapofylline (BG9928) reduced glomerulosclerosis in ZSF1 rats used as an animal model of HFpEF related to metabolic syndrome (Tofovic et al., 2016). Diabetic nephropathy is associated with reduced nitrergic vascular innervation, NO bioavailability and VEGF-NO uncoupling leading to 1) excessive endothelial cell proliferation, 2) stimulation of macrophage chemotaxis and 3) vascular smooth muscle cells growth, which ultimately causes glomerular hypertrophy and proteinuria (Nakagawa, 2007). Taking these findings together, it may well be that the renoprotective effect of BG9928 in preventing glomerulosclerosis may be undertaken by the A_2B_AR antagonism (Tofovic et al., 2016). Indeed, isolated renal fibroblasts treated with the A_2B_AR agonist, BAY-606583, increased the release of pro-inflammatory and pro-fibrotic mediators (Wilkinson et al., 2016). On the other hand, blockage of the A_2B_AR with MRS1754 significantly decreased albuminuria and improved the renal function in streptozotocin-induced diabetic mice; the antagonism of overexpressed A_2B_AR normalized VEGF-A upregulation in these animals while enhancing NO production and decreasing the occurrence of glomerulosclerosis lesions (Table 2) (Patel and Thaker, 2014).

In a recent study performed using a rat model of HF secondary to renal injury, the mineralocorticoid receptor antagonist, spironolactone, significantly up-regulated A_2A_AR expression, inhibited endothelial-to-mesenchymal transition and attenuated cardiorenal fibrosis, both *in vivo* and *in vitro* (Table 2) (Chen et al., 2019). These outcomes suggest the A_2A_AR as a potential therapeutic target for the cardiorenal syndrome (Chen et al., 2019). In addition to the anti-fibrotic role of the A_2A_AR, its ability to dilate the efferent arteriole may be renoprotective by reducing the glomerular pressure (Pandey et al., 2021). Long-term administration of the selective A_2A_AR agonist, CGS21680, reduces diabetes-induced glomerular hyperfiltration in streptozotocin-treated diabetic mice through a mechanism depending on NO production (Table 2) (Persson et al., 2015b). The anti-inflammatory role of A_2A_AR activation was reflected by a decrease in the number of infiltrating immune cells and by the reduction of urinary pro-inflammatory cytokines along with reduced albuminuria (Awad et al., 2006). These results were corroborated by a different research group using the same animal model, who proved that selective A_2A_AR activation with CGS21680 prevents proteinuria and glomerular damage by activating T regulatory cells, which results in reduced macrophage infiltration and TNF-α secretion (Table 2) (Persson et al., 2015a). Likewise, inhibition of ADK leading to adenosine overflow to the extracellular milieu had a protective role against renal failure in streptozotocin-induced diabetic mice by decreasing renal inflammation and oxidative stress lesions, as well as by restoring glomerular filtration and permeability (Table 2) (Pye et al., 2014). These effects were consistent with the reduction in renal macrophage infiltration, nuclear factor-κB phosphorylation and monocyte chemoattractant protein-1 excretion, along with increased endothelial NOS expression and MAPK phosphorylation (Pye et al., 2014).

In a model of hypertensive-diabetic nephropathy using spontaneously hypertensive rats treated with streptozotocin, the enzymatically-stable adenosine analogue, CADO, improved glucose metabolism (decreased hyperglycaemia and glycosuria), renal function (decreased proteinuria), and renal fibrosis (decreased glomerular collagen deposition), along with decreased renal oxidative stress (Table 2) (Patinha et al., 2020); authors implicated renal A_2A_AR activation in these findings, given that overexpression of this receptor, but of A_1_AR and A_2B_AR, was observed in superficial glomeruli and proximal and distal tubules of these animals’ kidneys (Patinha et al., 2020). The hypertension-inducing adenosine receptor antagonist, 1,3-dipropyl-8-sulfophenylxanthine, aggravated renal fibrosis, but did not alter the global metabolic status and renal function. This probably results from the selective preservation of tonic A_3_AR activation, given that downregulation of this receptor is considered protective against renal fibrosis (Patinha et al., 2020). Taken together, these results emphasize the protective role of adenosine in hypertensive-diabetic nephropathy through dynamic expression of its receptors, which may be fine-tuned by A_2A_AR upregulation and A_3_AR downregulation, thus prompting for a novel therapeutic target for this disease conditions (Patinha et al., 2020).

A_3_AR overexpression may be another target to abrogate progression of diabetic nephropathy in db/db leptin receptor-deficient mice, which is the most widely used animal model of type 2 DM normally presenting increased body weight, impaired glucose tolerance, and LV hypertrophy associated with HFpEF, but no significant changes in blood pressure. Chronic administration of LJ-2698 (a novel orally-active and highly selective A_3_AR antagonist) to these animals was more efficient in preventing diabetic nephropathy progression than the widely used AT1 receptor antagonist, losartan (Table 2) (Dorotea et al., 2018). Moreover, inhibition of renal lipid accumulation with increases in PGC1α, a key regulator of mitochondrial biogenesis, were obtained upon treating these animals with either LJ-2698 or losartan, further supporting the renoprotective effects of selective A_3_AR antagonism (Dorotea et al., 2018). Whether the combination of A_3_AR and AT1 receptor antagonists exerts synergic renoprotective effects requires investigation in future studies (Dorotea et al., 2018).

### Pulmonary Disease

Left heart disease-induced pulmonary hypertension (PH), the WHO group 2 of PH, tend to be more symptomatic and to have worse prognosis when compared to patients with left heart disease alone (Levine et al., 2019). Specifically, PH associated with diastolic dysfunction, also referred as PH secondary to HFpEF (PH-HFpEF) is the most common endophenotype attributable to left heart disease (Levine et al., 2019). Diastolic dysfunction coupled with left atria changes in HFpEF result in a passive backward transmission of increased LV filling pressure with consequent increases in pulmonary congestion (Levine et al., 2019). As aforementioned, studies in animal models of HFpEF induced by cardiac pressure overload revealed a putative beneficial role of adenosine in improving cardiac structure and function alterations and, thereby, attenuating cardiac dysfunction (Liao et al., 2003; Sabbah et al., 2013b) while decreasing pulmonary congestion (Liao et al., 2003). Major benefits of adenosine in this endeavor result from A_1_AR and A_2A_AR activation (Hamad et al., 2012; Quast et al., 2017; Zhou et al., 2020) associated or not with A_3_AR antagonism (Lu et al., 2008), which may also ameliorate symptoms and prognosis of HFpEF caused by cardiac pressure overload.

Adenosine levels in the pulmonary circulation are pathologically low in patients with WHO group 1 pulmonary arterial hypertension (PAH) as a consequence of local endothelial dysfunction and increased inactivation by ADA, possibly indicating that adenosine A_1_AR and A_2A_AR activation deficits unbalanced by inosine-mediated A_3_AR tone (Herman-de-Sousa et al., 2020) may contribute to maladaptive lung disease (Saadjian et al., 1999). In contrast to other vascular beds where adenosine has potent vasodilatory actions, in the pulmonary circulation, the nucleoside exerts dual opposing effects depending on the vascular tone (Cheng et al., 1996). Under low pressure conditions, adenosine fosters vasoconstriction through A_1_AR activation, whereas upon increasing the vascular tone, as observed in PH, the A_2_AR subtypes acquire more relevance to promote vasodilatation.

Among all adenosine receptor subtypes, the A_2A_AR gathered the most attention concerning PAH treatment. This assumption is based on the fact that A_2A_AR KO mice exhibit structural and functional abnormalities similar to PAH (Xu et al., 2011; Shang et al., 2015) and overexpress RhoA and ROCK proteins, which are known to be involved in pulmonary vascular remodelling and PAH pathophysiology (Table 1) (Shang et al., 2015). In mice with PAH induced by monocrotaline, activation of A_2A_AR reduced pulmonary endothelial dysfunction and vascular remodelling, while increasing pulmonary vasodilation and the LV stroke volume (Table 1) (Alencar et al., 2013; Alencar et al., 2014). The way adenosine builds its positive influence on RV hypertrophy and dysfunction may be indirectly related to the counteracting effects of the A_2A_AR on increased pulmonary resistance (Table 1) (Alencar et al., 2014) via NO-mediated synergism (Alencar et al., 2018; see *Pros and Cons of Adenosine A*_*1*_ *Receptor Manipulation in HFpEF*). Additionally, the anti-inflammatory actions of A_2A_AR agonists may improve cardiopulmonary homeostasis, as discussed above.

Immunolocalization studies demonstrated infiltration of the RV myocardium by A_2B_AR -positive fibroblasts and macrophages in rats with monocrotaline-induced PAH (Braganca et al., 2015). This result is compatible with adenosine being able to downregulate pro-inflammatory and pro-fibrotic stimuli, thus contributing to mechanical adaptation of RV in response to cardiac pressure overload (Braganca et al., 2015). Chronic obstructive pulmonary disease, a highly prevalent pulmonary comorbidity in patients with HFpEF, is also linked to PH development. Interestingly, chronic obstructive pulmonary disease patients with PH exhibit increased pulmonary vascular remodelling and mastocyte-induced airway hyperactivity associated with A_2B_AR overexpression (Table 1) (Karmouty-Quintana et al., 2013). Notwithstanding this, the role of the low affinity A_2B_AR in cardiopulmonary pathophysiology lacks comprehensive studies before inferring any beneficial effects from targeting this receptor to improve RV failure secondary to PAH (reviewed in (Bessa-Goncalves et al., 2018)). Besides controversial studies claiming deleterious effects of A_2B_AR in cardiopulmonary pathophysiology, others (fewer) emphasize some beneficial effects from A_2B_AR activation (Bessa-Goncalves et al., 2018).

Considering that most studies available so far about the role of the A_2B_AR in cardiopulmonary function rely on animal models of PAH, care must be taken before extrapolating the therapeutic effects of this receptor in PH-HFpEF. While the vast majority of PH-HFpEF patients develop HF secondary to pulmonary venous congestion (isolated post-capillary PH) (Levine et al., 2019), studies have shown that many patients with HFpEF also present with coexisting pulmonary vascular disease due to a metabolic syndrome-induced low grade inflammatory status (Ranchoux et al., 2019). Together with the control of actual lifestyle trend leading to increased prevalence of cardiometabolic diseases, development of novel therapeutic strategies towards combined pre- and post-capillary PH is an unmet clinical need (Ranchoux et al., 2019). Through its cardiovascular protective effects and role in metabolic diseases, together with aforementioned effects in PAH, adenosine signalling stands as a putative novel target to manage PH-HFpEF, providing that data from preclinical studies translate correctly to clinical trials in humans.

## Targeting Adenosine Signal Nuances in the Treatment of HFpEF

Adenosine handling, metabolism and signalling are implicated in cardiac remodelling and progression to HF (Table 3). Indeed, increased cardiac and plasma adenosine levels are hallmarks of severity in patients with HF, which might result from decreased cellular energy charge, shift to anaerobic metabolism, plasma membrane damage and decreased ADA activity (Funaya et al., 1997; Asakura et al., 2007). Excessive adenosine endogenous tone may lead to downregulation of adenosine receptor genes, namely A_2A_AR, A_2B_AR, and A_3_AR, in the failing heart (Funaya et al., 1997; Asakura et al., 2007). Increased endogenous levels of adenosine may reflect an adaptive mechanism to counteract adenosine signalling impairment in failing hearts (Funaya et al., 1997; Asakura et al., 2007). This is confirmed because increases in the myocardial concentration of adenosine resulting from inhibition of the nucleoside cellular uptake are protective against cardiac remodeling and *ß*-adrenergic dysfunction, thereby counteracting progression to HF (Chung et al., 1998).

**TABLE 3:** Pros and cons of targeting adenosine receptors, enzymes and metabolism in molecular pathways underlying heart failure with preserved ejection fraction and cardioprotection.

Adenosine signalling manipulation	Pharmacological approach		Refs
	Activation	Inactivation	
Protective role			
A_1_AR^a^	Decreased cardiac hypertrophy and dysfunction		Sabbah et al. (2013a); Liao et al. (2003); Puhl et al. (2016)
	Counteracted *ß*-adrenergic effects on cardiac remodelling through inhibition of cAMP^b^ stimulated-PKA^c^ activation and inhibition of noradrenaline release from cardiac nerves		Burgdorf et al. (2001); Meyer et al. (2001)
	Attenuated mitochondrial dysfunction by decreased opening rate of mitochondrial pores		Sabbah et al. (2013a)
	Change in energy substrate utilization with increased expression plasma membrane expression of GLUT^d^-1 and -4		Sabbah et al. (2013a)
	Ameliorated SERCA2a^e^ function and improved intracellular calcium handling		Sabbah et al. (2013a)
	Myocardial protection under ischemic preconditioning and ischemia		Reichelt et al. (2005); Morrison et al. (2006); Greene et al. (2016)
	Increased capillary density and oxygen diffusion distance		Sabbah et al. (2013a)
	Improved metabolic profile through regulating insulin sensitivity and glucose tolerance		Cheng et al. (2000); Johansson et al. (2008); Staehr et al. (2013)
	Attenuated kidney metabolic demand, tubular cell apoptosis and inflammatory reaction, along with a reduction of the glomerular filtration pressure		Lee et al. (2004); Xiong et al. (2018)
A_2A_AR	Endothelial dependent and independent coronary vasodilation		Berwick et al. (2010); Li et al. (1995); Li et al. (1998); Faria et al. (2006)
A_2B_AR	Angiogenic properties		Ahmad et al. (2013); Du et al. (2015)
A_2A_AR	Decreased cardiac inflammation, fibrosis, hypertrophy and dysfunction		Zhou et al. (2020)
	Enhanced intracellular calcium homeostasis		Hamad et al. (2012)
	Enhanced cardiac contractility		Dobson and Fenton (1997)
	Cardioprotection during early reperfusion after MI^f^		Jordan et al. (1997); Yang et al. (2006); Rork et al. (2008)
	Improved lipolysis, browning process of white adipose tissue, and insulin signalling; decreased levels of free fatty acids		de Oliveira et al. (2020)
	Decreased renal inflammation, fibrosis and oxidative stress		Awad et al. (2006); Persson et al. (2015a); Persson et al. (2015b); Chen et al. (2019); Patinha et al. (2020); Pandey et al. (2021)
	Attenuated glomerular hyperfiltration		
	Improved renal function		
	Decreased pulmonary vascular remodelling		Alencar et al. (2013); Alencar et al. (2014); Alencar et al. (2018)
	Attenuated right ventricle hypertrophy and dysfunction		
A_2B_AR	Attenuated cardiac fibrosis *in vitro*		Phosri et al. (2017); Phosri et al. (2018); Delaunay et al. (2019)
	Reduced infarct size under ischemic preconditioning		Eckle et al. (2007a)
	Decreased adipogenesis, increases lipolysis and improved insulin signalling		Gharibi et al. (2012); Peleli et al. (2015)
	Decreased pulmonary artery pressure and proliferation of smooth muscle fibers		Bessa-Goncalves et al. (2018)
	Stimulated angiogenesis Exerted cardioprotective effects during reperfusion phase after MI		Hinze et al. (2012)
			Ge et al. (2010); Hussain et al. (2014); Tian et al. (2015a)
A_3_AR	Increased plasma adenosine		Chung et al. (1998)
	Significantly improved LV^h^ filling and decreased pulmonary congestion		
	Attenuated *ß*-adrenergic dysfunction		
	Coronary vasodilation		Drury and Szent-Gyorgyi, (1929)
CD73		Increased cardiac inflammation, fibrosis, hypertrophy, and dysfunction	Xu et al. (2008); Quast et al. (2017)
		Increased infarct size under ischemic preconditioning	Eckle et al. (2007a)
ADK^i^		Attenuated the adenosine mediated anti-hypertrophic effect	Fassett et al. (2011); Fassett et al. (2019)
Deleterious role			
A_1_AR		Increased natriuresis and diuresis	Dittrich et al. (2007); Givertz et al. (2007), Voors et al. (2011)
A_2A_AR		Rescue the whole-body insulin sensitivity under high-sucrose diet	Sacramento et al. (2020)
A_2B_AR			
A_2B_AR		Attenuated the post-MI cardiac remodelling	Toldo et al. (2012); Zhang et al. (2014)
		Attenuated glomerulosclerosis and improved renal function	Patel and Thaker, (2014); Tofovic et al. (2016)
		Attenuation of pulmonary vascular remodelling and hypertension	Pye et al. (2014)
A_3_AR		Attenuated cardiac hypertrophy, fibrosis and oxidative stress and dysfunction	Lu et al. (2008)
		Decreased renal fibrosis, inflammation, oxidative stress, and lipid accumulation	Patinha et al. (2020)
		Decreased glomerular damage	
ADK		Epigenetic upregulation of pro-angiogenic genes and consequent endothelial proliferation and ischemia-induced angiogenesis	Xu et al. (2017b)
		Protection against the high fat diet induced insulin resistance and metabolic syndrome, in part due to its endothelial anti-inflammatory effects	Xu et al. (2019)
		Decreased renal inflammation and oxidative stress	Pye et al. (2014)
		Attenuated glomerular damage	
ADA^j^		Attenuated the diet-induced obesity and insulin resistance through decreased food intake	Cui et al. (2021)

AR^a^, Adenosine receptor; cAMP^b^, cyclic 5′-adenosine monophosphate; PKA^c^, protein kinase A; GLUT^d^, Glucose transporter type; SERCA^e^, sarco/endoplasmic reticulum Ca^2+^, ATPase; MI^f^, Myocardial infarction; ENT^g^, Equilibrative nucleoside transporters; LV^h^, Left ventricle; ADK^i^, Adenosine kinase; ADA^j^, Adenosine deaminase.

As already outlined, all adenosine receptor subtypes may be involved, in one way or another, in the pathophysiology of HFpEF and related comorbidities (Figures 2, 3). Data indicate that A_1_AR provides cardioprotective effects due to reversal of cardiac hypertrophy/remodelling, improvement of mitochondrial function, enhancement of SERCA2a activity and Ca^2+^ handling, increases in capillary density, modifications in substrate utilization, and enhancements in skeletal muscle performance. In addition, A_1_AR activation has been associated to anti-ischemic properties by decreasing catecholamine release and *ß*-adrenergic overactivation (Albrecht-Kupper et al., 2012; Greene et al., 2016). Activation of A_1_AR also improves the metabolic profile (de Oliveira et al., 2020) and attenuates renal metabolic demand and glomerular filtration pressure (Pandey et al., 2021). It is, therefore, not surprising that the most expressed adenosine receptor in the heart, the A_1_AR, has been the most widely studied receptor in cardiovascular, renal and metabolic diseases, both at preclinical and clinical levels (Greene et al., 2016; Borah et al., 2019; Jacobson et al., 2019; Shah et al., 2019) (for details, see *Pros and Cons of Adenosine A*_*1*_ *Receptor Manipulation in HFpEF*). Notwithstanding this, the anti-adrenergic cardioprotective effects of the A_1_AR may be partially counteracted by A_2A_AR activation (Chandrasekera et al., 2010), as detected in cardiac pressure overload (Meyer et al., 2001) and hypertensive animals (Tang et al., 1998).

Data from preclinical studies ascribed cardioprotective roles to the A_2A_AR subtype. Activation of A_2A_AR attenuates cardiac, renal and pulmonary damage due to its ability to counteract inflammation, in addition to its vasodilation and angiogenic properties; interestingly, this receptor also mediates increases in cardiac inotropism without interfering with Ca^2+^ homeostasis (Headrick et al., 2013). Positive cardiac repercussions against hypertensive remodelling resulting from A_2A_AR activation may be owe to amelioration of insulin resistance associated with its effects on brown adipocytes and inflammation (de Oliveira et al., 2020; Zhou et al., 2020). Controversy still exists regarding the impact of the A_2A_AR on the whole body insulin sensitivity, since antagonism of this receptor in skeletal muscles attenuated whole-body insulin resistance (Sacramento et al., 2020). Like that observed for the A_1_AR subtype, the anti-adrenergic effect of the A_2A_AR is also impaired in hypertensive animals (Tang et al., 1998). The chronic use of selective A_2A_AR agonists is also not free of cardiovascular side effects, like arterial hypotension and tachycardia. To avoid residual actions on other adenosine receptor subtypes, a new era of nucleoside-5′-monophosphates as prodrugs of selective A_2A_AR activators have been designed, which require ecto-5′-nucleotidase/CD73 activation in close proximity to receptor sites providing that the adenosine forming enzyme is available and functional (El-Tayeb et al., 2009; Flogel et al., 2012).

Conflicting evidence exists regarding A_2B_AR-mediated cardioprotection, as well as its pulmonary effects (Table 3) (Pye et al., 2014). While activation of A_2B_AR may counteract fibrosis (Phosri et al., 2017; Phosri et al., 2018; Delaunay et al., 2019) and ischemic preconditioning (Eckle et al., 2007a) in the heart, other studies suggest that this low affinity adenosine receptor may be deleterious in ischemic cardiac remodelling (Toldo et al., 2012; Zhang et al., 2014), as well as in renal fibrosis (Patel and Thaker, 2014; Tofovic et al., 2016). Like the A_2A_AR, the action of the A_2B_AR in DM and obesity is also controversial (Sacramento et al., 2020). The same occurs regarding the least expressed A_3_AR in the heart (Table 3) (Borea et al., 2018). The A_3_AR may be cardioprotective against reperfusion (Ge et al., 2010; Hussain et al., 2014; Tian et al., 2015a) by promoting angiogenesis (Hinze et al., 2012), but it may aggravate pathological structural changes in the heart (Lu et al., 2008) and kidney (Patinha et al., 2020).

Manipulation of endogenous adenosine inactivation through cellular uptake and/or ADA may be a valuable strategy to increase the extracellular concentration of the nucleoside and, thereby, its receptors activation. Inhibition of ENT by dipyridamole attenuates cardiac remodelling and LV dysfunction, prevents *ß*-adrenergic dysfunction and induces coronary vasodilation (Drury and Szent-Gyorgyi, 1929; Chung et al., 1998). Downregulation of ADA activity counteracts diet-induced obesity and insulin resistance through decreases in food intake (Cui et al., 2021).

Regulating intracellular adenosine concentration by manipulating ADK, a key intracellular enzyme that is responsible for maintaining low intracellular adenosine levels, thus favoring extracellular adenosine uptake by the cells, may also be a putative strategy to increase the extracellular levels of the nucleoside (Table 3). As a matter of fact, inhibition of ADK may be cardioprotective due to increases in the extracellular levels of the adenosine, which stimulates angiogenesis, promotes insulin sensitivity, and attenuates inflammation and oxidative stress (Pye et al., 2014; Xu et al., 2017b; Xu et al., 2019); contrariwise, when this enzyme is fully operative reduction of the extracellular levels of the nucleoside may be deleterious as it abrogates the anti-hypertrophic effect of adenosine (Fassett et al., 2011; Fassett et al., 2019).

### Pros and Cons of Adenosine A_1_ Receptor Manipulation in HFpEF

As previously mentioned, the A_1_AR has been the most widely studied adenosine receptor in the cardiovascular system, both at preclinical and clinical levels. Indeed, activation of A_1_AR exerts cardioprotective properties that go beyond the preservation of cardiac structure and metabolism, encompassing regulatory metabolic benefits. In this context, several A_1_AR agonists have been tested in clinical trials for type 2 DM. The first clinical trials using A_1_AR full agonists, ARA and GR79236, were successful in improving insulin sensitivity, but failed their endpoints due to undesirable cardiovascular side effects (Jacobson et al., 2019). As a matter of fact, the clinical use of A_1_AR full agonists may present some limitations related to off-target side effects, either cardiac (bradycardia up to atrioventricular block, negative dromotropy and inotropy) and extra-cardiac (functional depression of the central nervous system and reduction of glomerular filtration rate due to vasoconstriction of afferent arteriole) (Albrecht-Kupper et al., 2012; Greene et al., 2016). Another potential limitation of chronic administration of A_1_AR full agonists is receptor desensitization (Albrecht-Kupper et al., 2012). To overcome these limitations partial A_1_AR agonists have been produced and tested in clinical trials. These compounds exhibit a more favorable haemodynamic profile with minimal side effects in terms of heart rate, atrioventricular conduction, blood pressure and renal function, along with no evidence of adenosine-mediated negative inotropism (Albrecht-Kupper et al., 2012; Greene et al., 2016), which is in agreement with our prediction that adenosine acting via the A_1_AR is a chronoselective atrial depressant (Braganca et al., 2016). One of such compounds, GS-9667 (previously known as CVT-3619, a partial agonist that selectively binds to A_1_AR), reduced plasma free fatty acids and was well-tolerated both in healthy non-obese and obese subjects, without showing any signs of receptor desensitization or rebound problems on suspension (Table 2) (Staehr et al., 2013). The antilipidemic effect of this compound is not surprising because A_1_AR are highly more abundant in adipocytes than in the atrioventricular node (Wu et al., 2001; Liang et al., 2002).

Controversy still exists regarding the putative cardiorenal protection associated with A_1_AR modulation. The ability of A_1_AR antagonists to induce natriuresis without compromising the glomerular filtration rate suggests that this approach may be beneficial to control volume overload disorders, such as HF (Gottlieb et al., 2002). The selective A_1_AR antagonist, rolofylline, increased diuresis in patients with acute and chronic HF, with enhancements of the renal plasma flow also observed in the latter (Table 2) (Dittrich et al., 2007; Givertz et al., 2007). However, in the large phase III clinical trial PROTECT (Placebo-Controlled Randomized Study of the Selective A_1_ Adenosine Receptor Antagonist Rolofylline for Patients Hospitalized with Acute Decompensated Heart Failure and Volume Overload to Assess Treatment Effect on Congestion and Renal Function), rolofylline reduced fluid retention, but did not prevent the worsening of renal dysfunction in acute HF patients (Voors et al., 2011). This failure may be attributed to disproportionate diuresis associated with escalating doses of rolofylline, which might have offset the ability of this drug to preserve renal filtration rate and kidney blood supply (Voors et al., 2011). In fact, despite the putative benefits in controlling hypertension and volume overload, inhibition of the A_1_AR activity may be detrimental for glomerular architecture and function, as it increases intraglomerular pressure by antagonizing preglomerular vasoconstriction operated by A_1_AR (Tofovic et al., 2016). Thus, considering its cardioprotective and metabolic roles, as well as its ability to reduce intraglomerular pressure, A_1_AR agonists may stand as promising medications for treatment of the chronic cardiorenal syndrome.

Partial A_1_AR agonists were also tested in clinical trials for HF in patients presenting reduced or preserved ejection fraction (Voors et al., 2018). Data from two small pilot studies demonstrate that the novel orally-available partial A_1_AR agonist, neladesonon, seems to be safe with no atrioventricular, neurological, and renal side effects in patients with HF with reduced ejection fraction (Voors et al., 2017), but no beneficial early changes were observed in the cardiac function (Table 1) (Voors et al., 2017). Data from a phase II double-blinded placebo-controlled clinical trial (PANACHE - The Partial AdeNosine A_1_ receptor agonist in patients with Chronic Heart failure and preserved Ejection fraction), designed to explore the effects of neladesonon on exercise capacity among 300 patients with HFpEF, has been recently provided (Table 1) (Shah et al., 2019). Neladesonon did not have a significant impact on exercise capacity. This was interpreted as consequence of a lower than normal prevalence of coronary artery disease in HFpEF patients, together with a higher number of patients using *ß*-blockers that may abrogate the beneficial antiadrenergic effects of A_1_AR agonists (Shah et al., 2019). Although the results were disappointing, a low incidence of major adverse effects was observed in patients under neladesonon treatment (Shah et al., 2019).

The aforementioned data suggest that before translating the putative preclinical benefits of manipulating the adenosinergic signals to the therapeutic armamentarium, one must keep in mind that adenosine production and/or inactivation, receptor subtype expression dynamics, and downstream intracellular messenger pathways might change in a moment-to-moment basis depending on the tissue, pathological situation and stage of disease condition. Another pitfall in our current knowledge is due to the relatively complex pathophysiology of HFpEF owing to a limited number of animal models that reflect entire features of the human pathology; this constrain, further challenges translation of preclinical data to clinical settings (Conceicao et al., 2016). Notwithstanding this, the beauty of targeting the adenosinergic system is that it can differentially control not only heart structure and function abnormalities, but also most of the co-morbidities concurring with HFpEF in terms of precipitating causes and/or aggravating factors.

## Conclusion

Taken together, these preclinical and clinical evidence shed some lights on the adenosine role in molecular mechanisms and comorbidities related to HFpEF. Adenosine was found to counteract cardiac pathophysiological mechanisms implicated in this clinical syndrome, namely cardiac inflammation and microvascular dysfunction, especially via A_2_ARs, in addition to extracellular and cellular structural abnormalities, and energy metabolism and Ca^2+^ handling, being these effects more under A_1_AR and A_2_AR control. Consequently, adenosine also counteracts cardiac remodelling and related diastolic dysfunction, the pathological hallmark of HFpEF clinical syndrome. Considering the new paradigm of a systemic microvascular dysfunction as a perpetuating factor for myocardial dysfunction, adenosine receptors, enzymatic machinery and downstream intracellular messenger pathways surge as promising treatment targets, in part because they are also implicated in a wide range of pathophysiological processes related to HF comorbidities, such as diabetes and metabolic syndrome, along with kidney and pulmonary dysfunction.
